# Targeted Proteolysis of Plectin Isoform 1a Accounts for Hemidesmosome Dysfunction in Mice Mimicking the Dominant Skin Blistering Disease EBS-Ogna

**DOI:** 10.1371/journal.pgen.1002396

**Published:** 2011-12-01

**Authors:** Gernot Walko, Nevena Vukasinovic, Karin Gross, Irmgard Fischer, Sabrina Sibitz, Peter Fuchs, Siegfried Reipert, Ute Jungwirth, Walter Berger, Ulrich Salzer, Oliviero Carugo, Maria J. Castañón, Gerhard Wiche

**Affiliations:** 1 Department of Biochemistry and Cell Biology, Max F. Perutz Laboratories, Centre for Molecular Biology, University of Vienna, Vienna, Austria; 2 Department of Medicine I, Institute of Cancer Research, Medical University of Vienna, Vienna, Austria; 3 Department of Structural and Computational Biology, Max F. Perutz Laboratories, University of Vienna, Vienna, Austria; 4 Department of General Chemistry, University of Pavia, Pavia, Italy; Northwestern University, United States of America

## Abstract

Autosomal recessive mutations in the cytolinker protein plectin account for the multisystem disorders epidermolysis bullosa simplex (EBS) associated with muscular dystrophy (EBS-MD), pyloric atresia (EBS-PA), and congenital myasthenia (EBS-CMS). In contrast, a dominant missense mutation leads to the disease EBS-Ogna, manifesting exclusively as skin fragility. We have exploited this trait to study the molecular basis of hemidesmosome failure in EBS-Ogna and to reveal the contribution of plectin to hemidesmosome homeostasis. We generated EBS-Ogna knock-in mice mimicking the human phenotype and show that blistering reflects insufficient protein levels of the hemidesmosome-associated plectin isoform 1a. We found that plectin 1a, in contrast to plectin 1c, the major isoform expressed in epidermal keratinocytes, is proteolytically degraded, supporting the notion that degradation of hemidesmosome-anchored plectin is spatially controlled. Using recombinant proteins, we show that the mutation renders plectin's 190-nm-long coiled-coil rod domain more vulnerable to cleavage by calpains and other proteases activated in the epidermis but not in skeletal muscle. Accordingly, treatment of cultured EBS-Ogna keratinocytes as well as of EBS-Ogna mouse skin with calpain inhibitors resulted in increased plectin 1a protein expression levels. Moreover, we report that plectin's rod domain forms dimeric structures that can further associate laterally into remarkably stable (paracrystalline) polymers. We propose focal self-association of plectin molecules as a novel mechanism contributing to hemidesmosome homeostasis and stabilization.

## Author Summary

Hemidesmosomes are specialized protein complexes that promote anchorage of the basal keratinocyte cell layer of the epidermis to the underlying dermis. They provide tissue integrity and resistance to mechanical forces. When hemidesmosomes do not function properly, skin blistering ensues in response to mechanical trauma. Plectin is an essential component of hemidesmosomes. Humans carrying recessive mutations in the plectin gene most frequently develop multisystem disorders, where in addition to skin other tissues are also affected. However, there is a unique dominant plectin mutation, which leads to the disease epidermolysis bullosa simplex Ogna (EBS-Ogna), affecting skin exclusively. Because of that, EBS-Ogna is an exceptional system to study the contribution of plectin to hemidesmosome function. We have generated an EBS-Ogna mouse model that mimics the human disease. Using this model, we have learned that selective degradation of hemidesmosome-associated plectin isoform 1a by proteases activated specifically in keratinocytes results in reduced numbers and dysfunction of hemidesmosomes. In contrast, plectin-1c, another plectin isoform expressed in keratinocytes, is not degraded. Moreover, we find that plectin dimers can oligomerize via their long coiled-coil rod domain, a process likely to be instrumental in maintenance of hemidesmosome integrity. These findings highlight the importance of plectin-1a for hemidesmosome function.

## Introduction

The cells of the basal layer of stratified epithelia are firmly attached to the underlying basement membrane through specialized multiprotein complexes called hemidesmosomes (HDs). In skin, the hemidesmosomal protein complex provides stable adhesion of the epidermis to the underlying dermis and ensures resistance to mechanical stress. HDs in skin contain the two cytolinker family members plectin and BPAG1e, integrin (ITG) α6β4, type XVII collagen BPAG2 (BP180), and tetraspanin CD151 [1].

Plectin is a highly versatile cytolinker protein that cross-links different types of intermediate filaments (IFs), connects them to the other cytoskeletal networks, and anchors them to the subplasma membrane cytoskeleton and to plasma membrane–cytoskeleton junctional complexes [2], [3]. Its versatility stems in part from a variety of alternatively spliced transcripts that encode different isoforms, varying in short N-terminal sequences that determine their cellular targeting [4], [5]. In the skin, as well as in cultured keratinocytes, plectin isoform 1a (P1a) is specifically recruited to HDs, while other isoforms, including P1c, are more prominent at cell-cell borders and interior cellular sites [6]. With an N-terminal actin-binding domain (ABD) [7], which serves also as an ITGβ4-binding site [8], and a C-terminal IF-binding site, plectin is instrumental in the physical anchorage of keratin IFs at the HD complex [8]–[10]. Whereas, in skeletal muscle, different isoforms (P1f and P1d) integrate myofibers by specifically targeting and linking desmin IFs to Z-disks and costameres [11], [12]. The concept that different plectin isoforms have distinct tissue-and cell type-specific functions recently received strong support from a report showing that loss of plectin 1f in humans affected only skeletal muscle but not skin [13].

Most mutations in the plectin gene are inherited in an autosomal-recessive fashion resulting in EBS-MD (EBS with muscular dystrophy, MIM:226670), EBS-PA (EBS with pyloric atresia, MIM:612138), and EBS-CMS (EBS with congenital myasthenia [14]). In contrast, EBS-Ogna (MIM:131950) is caused by an autosomal dominant mutation. This rare mutation is a heterozygous C>T transition at cDNA position 5998 (RefSeq NM_000445.3, GenBank) leading to a p.Arg2000Trp (formerly p.Arg2110Trp) substitution [3], [15] in the central rod domain (RD) separating the N- and C-terminal globular domains of plectin. The only known function of this domain is to mediate dimerization of plectin via formation of a coiled-coil RD. Common clinical symptoms of EBS-Ogna include a generalized bruising tendency and blistering of the skin, predominantly on hands and feet [15]. Histologically, EBS-Ogna skin blisters originate in the deepest areas of the basal cell cytoplasm, immediately above HDs, and the basal keratinocyte cell layer lacks anti-plectin immunoreactivity [15], [16]. In contrast, no differences in plectin expression were detected in skeletal muscle and EBS-Ogna patients do not develop any muscular pathology [15]. Structurally, Ogna-HDs display mostly normal intracellular attachment plates, but the insertion of keratin IFs into these plates is impaired [15].

The fact that a single amino acid substitution in a protein as large as plectin (>4500 residues), in a subdomain that is shared between the isoforms, leads to an autosomal-dominant disease with exclusive skin manifestation, elevates the interest in this mutation, as it may reveal novel features of the protein that are likely to be HD-related. To investigate the pathogenesis of the Ogna mutation and the role of plectin in HD stabilization we combined a knock-in approach with ex vivo and in vitro analyses. In this work we show that plectin^Ogna/+^ mice mimic key features of EBS-Ogna, resulting from impaired HD formation, due to proteolytic cleavage of HD-associated P1a by proteases specifically activated in the epidermis. Furthermore, we discovered that plectin's RD can oligomerize via lateral association, providing new insights into HD homeostasis and stabilization of the skin.

## Results

### EBS-Ogna mouse lines mimic the human disease

To investigate skin-specific traits of plectin molecules we generated a knock-in mouse line (*Plec* ^Ogna/+^) that carried the Ogna mutation on one of its plectin alleles. Details about the targeting strategy, analysis of ES clones, PCR genotyping of offsprings, and transcript expression patterns in tissues and primary keratinocytes are given in Figure S1. To study gene dosage effects, we also bred homozygous knock-in mice (*Plec* ^Ogna/Ogna^). Both, *Plec* ^Ogna/+^ and *Plec* ^Ogna/Ogna^ mice were viable, fertile and did not differ from wild-type littermates in size, postnatal development, reproduction rates, or life span.

**Figure 1:**
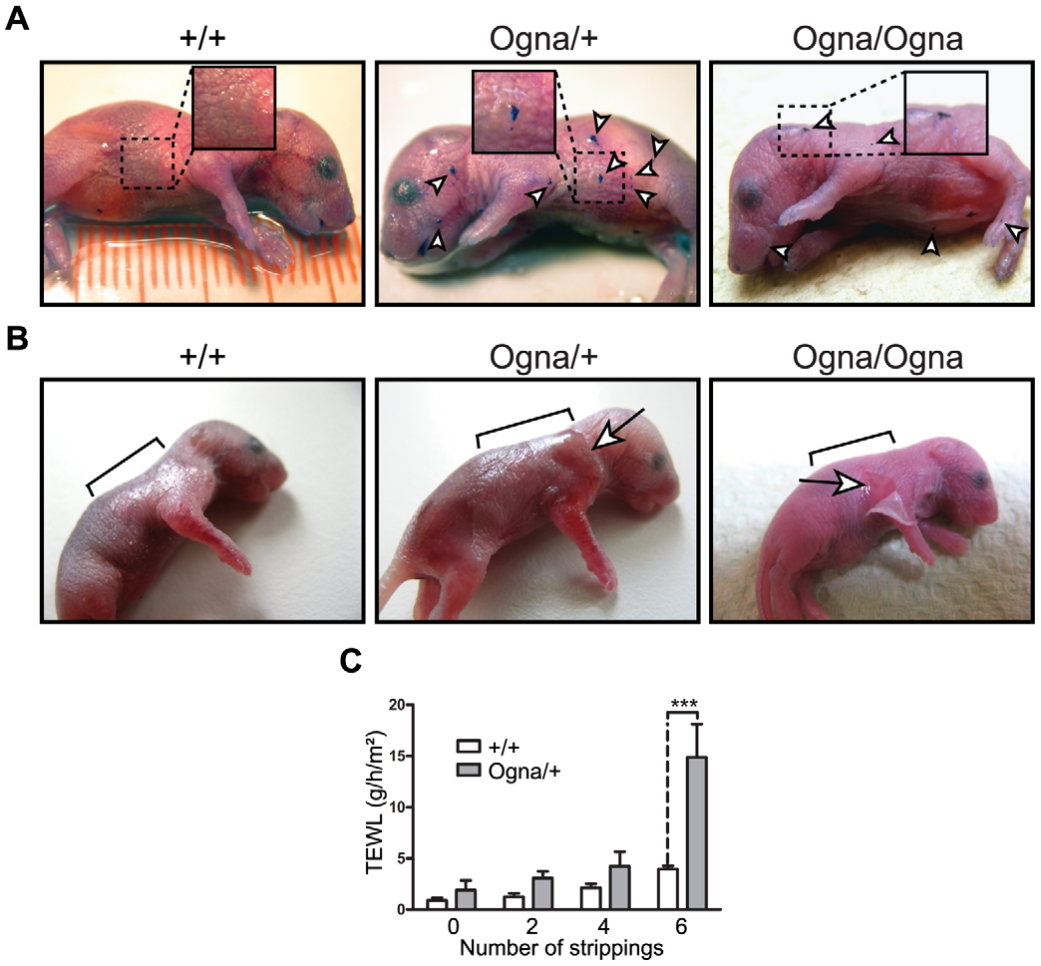
Phenotypical analysis of Ogna mice. (A) Toluidine blue dye penetration assays. Note localized breaches (arrowheads) of the skin barrier in 1-day-old *Plec* ^Ogna/+^ and *Plec* ^Ogna/Ogna^ mice, not noticeable in *Plec* ^+/+^ littermates. (B) Epidermal detachment after tape stripping. Epidermal exfoliation was visible after 6 consecutive tape strippings of 1-day-old *Plec* ^Ogna/+^ mice using D-Squame disks (CuDerm corporation, Dallas, TX) (arrow), whereas for *Plec* ^Ogna/Ogna^ mice, 2–3 tape strippings were usually enough for epidermal detachment (arrow). Brackets mark areas to which tape stripping was applied. (C) Transepidermal water loss (TEWL) after 6 consecutive tape strippings was strongly increased in 1-day-old *Plec* ^Ogna/+^ mice, compared to their *Plec* ^+/+^ littermates. Data are shown as mean values ±95% CI (n = 6). *** P<0.001 (two-way ANOVA with Bonferroni post test).

*Plec* ^Ogna/+^ and *Plec* ^Ogna/Ogna^ mice were born without any obvious skin defects. However, thorough inspection of their skin under a stereo microscope revealed the presence of small epidermal lesions, most frequently on the head, legs, and back skin, which could clearly be visualized when stained with toluidine blue [17] (Figure 1A). To test the resistance of the epidermis to mild mechanical stress we applied tape stripping followed by measurements of transepidermal water loss (TEWL) [17] (Figure 1B and 1C). Six consecutive tape strippings applied to the back skin of 1-day-old *Plec* ^Ogna/+^ mice caused epidermal exfoliation (Figure 1B), resulting in a ∼3.5-fold increase in TEWL (Figure 1C), whereas in the case of their *Plec* ^+/+^ littermates, the same treatment only moderately increased TEWL (Figure 1C). Skin fragility of comparable severity was also observed in newborn *Plec* ^Ogna/Ogna^ mice, albeit 2–3 tape strippings were usually enough to detach the epidermis from the underlying dermis (Figure 1B). A few days after birth, pups of all genotypes developed fur that served as a natural skin protection, with no pathological alteration becoming noticeable thereafter.

### Altered morphology and decreased numbers of HDs in Ogna epidermis

**Figure 2:**
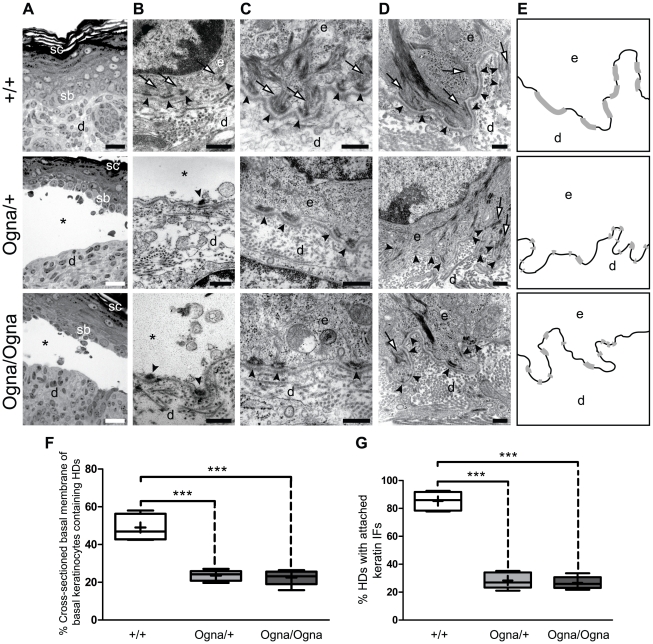
Histological and ultrastructural abnormalities of Ogna mouse skin. (A) Epoxy resin-embedded and toluidine blue-stained skin sections from 1-day-old mice. Asterisks indicate trauma-induced blister formation between stratum basale (sb) and dermis (d), not observed in *Plec* ^+/+^ mice. sc, stratum corneum. (B) Transmission electron microscopy of skin lesions. Arrowheads indicate intact HDs with attached keratin filament bundles (arrows) aligned along the basal cell membrane of basal keratinocytes in wild-type skin, and remnants of HDs lacking keratin filaments at blister (*) floors in mutant skin. (C,D) High magnification electron micrographs of intact skin sections of newborn (C) and adult (D) mice. (E) Outlines of HDs (grey bars) distributed along the basal cell membrane of basal keratinocytes (black lines) in panels D. Note reduced numbers and smaller sizes of HDs (arrowheads in C and D) in mutant, compared to *Plec* ^+/+^ skin; also, few HDs in mutant skin have attached keratin filament bundles, whereas thick bundles of filaments attached to HDs are dominant in *Plec^+/+^* skin (arrows in C and D). d and e (B–E), dermis and epidermis, respectively. Bars, 20 µm (A); 500 nm (B–D). (F,G) Morphometric analysis of HD numbers (average percentage of cross-sectioned basal cell membrane of basal keratinocytes containing HDs) (F) and keratin filament attachment (G) in adult mouse skin. A total length of 55–60 µm of basal cell membrane of basal keratinocytes was analyzed in electron micrographs of foot pad skin sections from wild-type and mutant littermates (n = 5, total numbers of HDs scored: *Plec^+/+^*, 617; *Plec^Ogna/+^*, 590; *Plec^Ogna/Ogna^*, 605). Box and whisker plots indicate the median (middle line in the box), the mean (small crosses), 25^th^ percentile (bottom line of the box), 75^th^ percentile (top line of the box), and 2.5^th^ and 97.5^th^ percentiles (whiskers). *** P<0.001, one-way ANOVA with Tukey post test for multiple comparisons.

The histological analysis of skin lesion biopsies from 1-day-old *Plec* ^Ogna/+^ and *Plec* ^Ogna/Ogna^ mice revealed a separation of the epidermis from the dermis at the level of basal keratinocytes (Figure 2A). In nonlesional areas, no differences in the organization of mutant and wild-type epidermis were noticed (data not shown). Transmission electron microscopy of lesional skin revealed that epidermis–dermis separation occurred just above the HDs of the basal cells, leaving the resulting blister floor covered by cytoplasmic debris including fragments of HDs (Figure 2B, arrowheads). Cytolysis at a level just above HDs is a characteristic diagnostic feature of plectin-associated EBS [9], [18], [19]. HDs in skin of newborn *Plec* ^Ogna/+^ and *Plec* ^Ogna/Ogna^ mice displayed normal ultrastructure with well defined inner and outer plates, except that the insertion of keratin IF bundles into HD inner plates appeared strongly diminished (Figure 2C). Furthermore, the numbers of HDs appeared to be reduced, irrespective of the site the skin sample was taken from. The vast majority of visualized HDs possessed inner plate structures, without noticeable differences among the three genotypes (data not shown). HDs in skin samples from adult mutant mice displayed similar morphological alterations (Figure 2D). Quantitative analyses showed that the percentage of cross-sectioned basal cell membrane of basal keratinocytes containing HDs was reduced by ∼45% in mutant mice compared to their *Plec* ^+/+^ littermates (Figure 2F). In addition, in adult as well as newborn mice, a clear trend towards smaller HDs and a strong reduction in keratin IF–HD attachment was observed (Figure 2G, and data not shown). Interestingly, both traits, number and morphology of HDs, were similarly affected regardless of the allelic state of the mutation (Figure 2F and 2G), indicating that a single copy of the mutation suffices to impair HD function. In contrast, desmosomal structures of mutant skin showed no ultrastructural abnormalities (data not shown).

### Altered protein expression in Ogna keratinocytes but not in muscle cells

**Figure 3:**
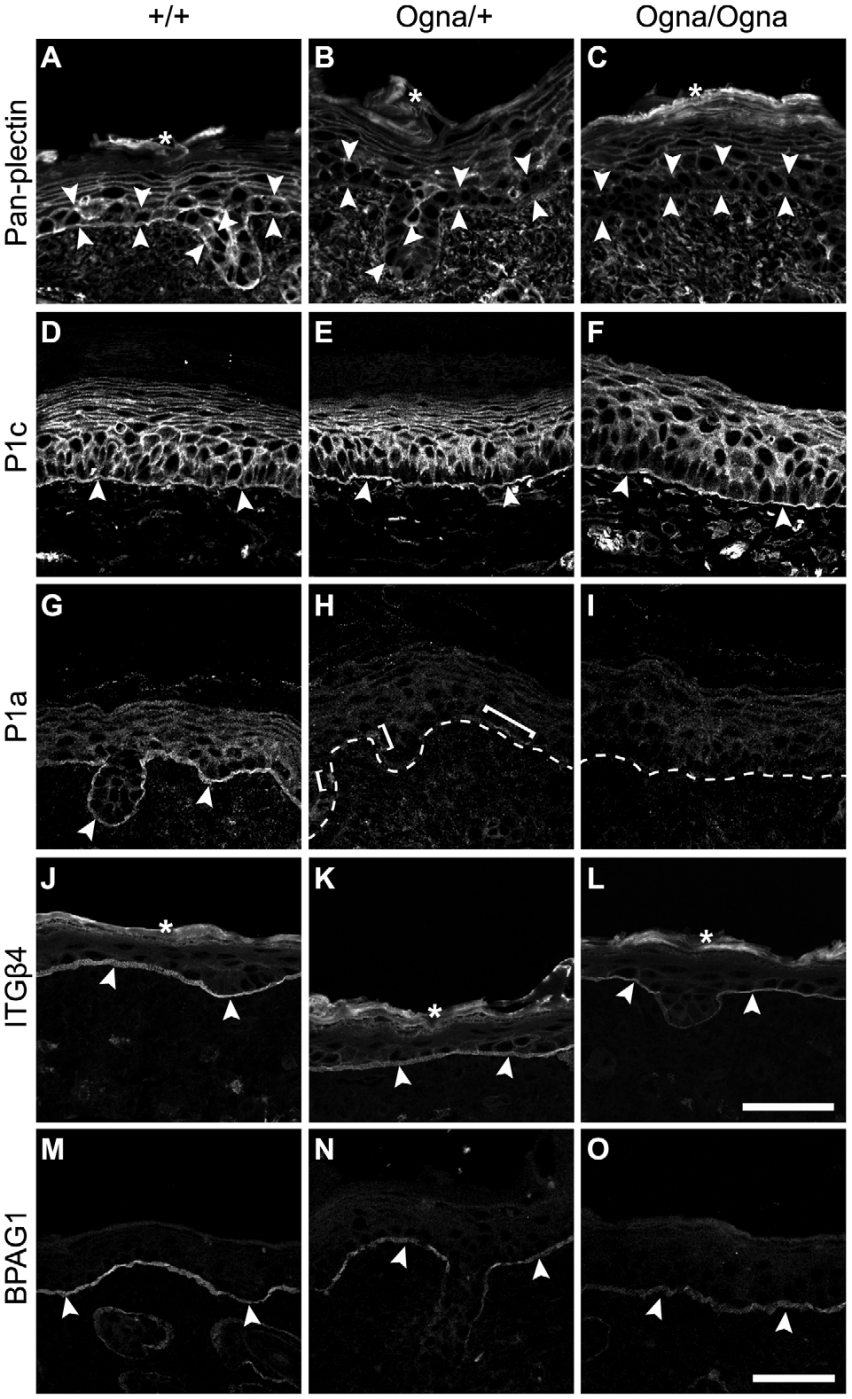
Immunolocalization of HD proteins on frozen leg skin sections from 1-day-old wild-type and mutant mice. (A–C) Visualization of plectin without isoform discrimination using mAb 10F6 (pan-plectin). Note downregulation of plectin expression in basal keratinocytes (opposing arrowheads) in mutant skin (B,C). (D–I) Visualization of P1c and P1a using isoform-specific antibodies. Expression and localization of P1c is comparable between all three genotypes (D–F). P1a is predominantly expressed at the basal cell membrane of basal keratinocytes in *Plec* ^+/+^ skin (G, arrowheads), but is absent in mutant epidermis, except for a few P1a-positive patches (H, brackets) remaining in *Plec* ^Ogna/+^ skin. Dashed lines, dermo-epidermal border. (J–L) Expression of ITGβ4 at the basal cell membrane of basal keratinocytes (arrowheads) is unaltered in *Plec* ^Ogna/+^ skin (K), but slightly downregulated in *Plec* ^Ogna/Ogna^ cells (L). (M–O) The BPAG1 signal along the basal cell membrane of basal keratinocytes (arrowheads) is more discontinuous in mutant compared to *Plec* ^+/+^ skin. Asterisks, unspecific labeling of stratum corneum in A–C and J–L. Bars in L (A–L) and O (M–O), 50 µm.

To examine the expression of major HD proteins, we subjected frozen skin sections from 1-day-old and adult mice to immunofluorescence microscopy. First we used antibodies to plectin that have routinely been applied in the diagnosis of plectin-associated EBS, including EBS-Ogna, and that do not discriminate between isoforms [15], [16], [20], [21]. In epidermis of newborn *Plec* ^+/+^ mice we detected prominent staining of the dermo–epidermal borderline, along with less pronounced staining of all epidermal keratinocyte membranes, except those of stratum corneum cells (Figure 3A). However, in the corresponding mutant tissues, anti-plectin immunoreactivity was found to be strongly reduced in basal keratinocytes (particularly along their basal cell membrane), whereas it was just slightly diminished in suprabasal cell layers (Figure 3B and 3C). Using isoform-specific antibodies, we found expression of P1c, the major plectin variant expressed in the epidermis (see below), to be well preserved in the mutant tissues (Figure 3D–3F). In striking contrast, no expression of plectin's HD-associated isoform P1a was observed in the epidermis of mutant mice (Figure 3H and 3I), except for a few P1a-positive patches in *Plec* ^Ogna/+^ epidermis (Figure 3H). The expression of the HD plectin-docking protein ITGβ4 in all three genotypes was found confined to the basal cell membrane of basal keratinocytes (Figure 3J–3L), but appeared to be slightly reduced in *Plec* ^Ogna/Ogna^ epidermis (Figure 3L). The signal intensity for HD-associated BPAG1 in mutant keratinocytes was retained, although its appearance was more patchy compared to wild-type samples (Figure 3M–3O), while the staining of keratin 5 (K5) was unaltered (Figure S2). In skin samples from adult *Plec* ^Ogna/+^ mice, expression of P1a was highly reduced, and hardly if at all detectable in *Plec* ^Ogna/Ogna^ mice, whereas in both mutants the expression levels of P1c, BPAG1, ITGβ4, and K5 were comparable to those of wild-type mice (data not shown).

**Figure 4:**
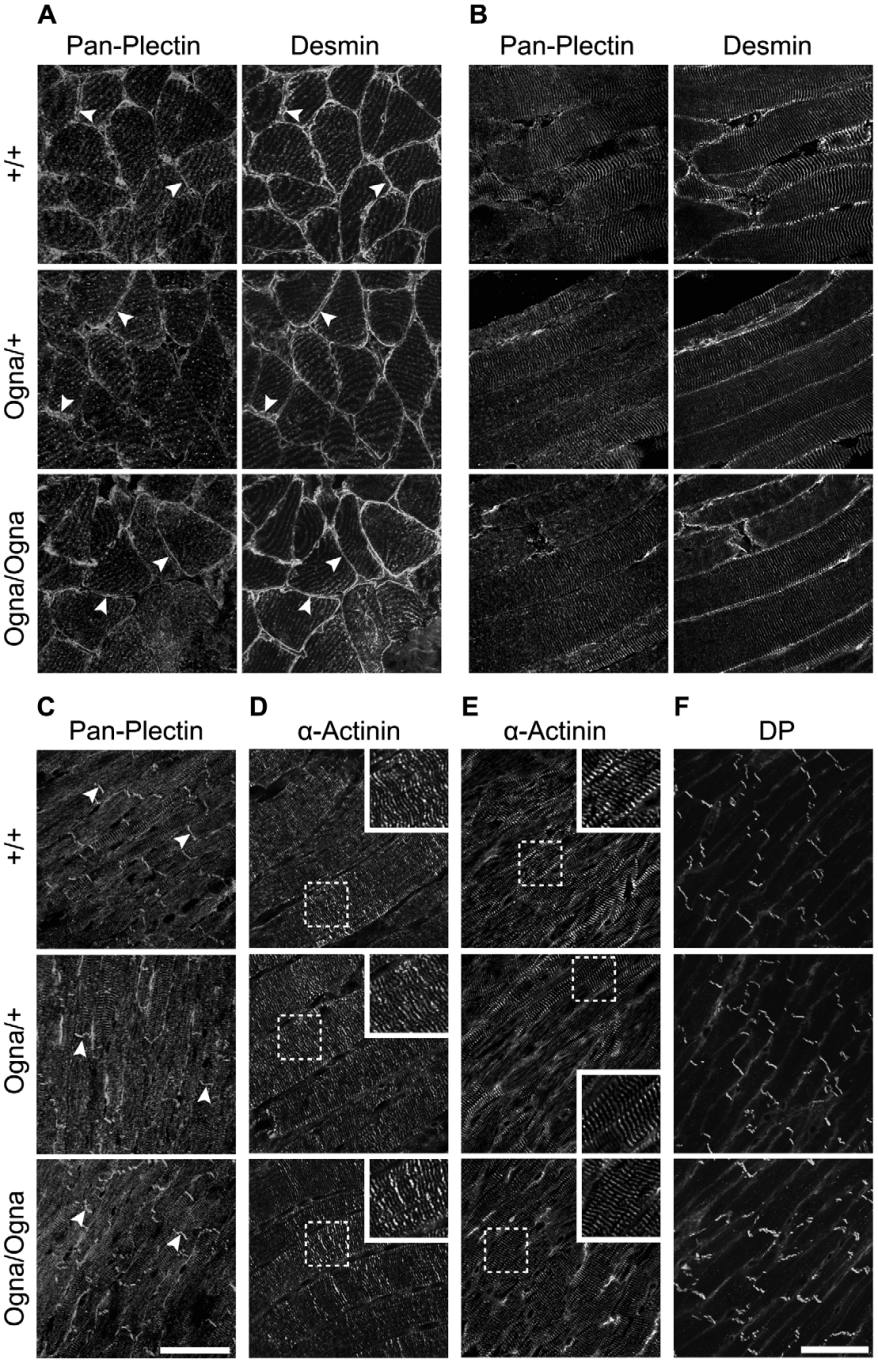
Normal plectin expression and contractile apparatus organization in muscle tissues of Ogna mice. (A,B) Cross sections (A) and longitudinal sections (B) of soleus muscle were double immunolabeled for plectin and desmin. Note, similar expression patterns of plectin and desmin at the sarcolemma (arrowheads) of *Plec* ^+/+^ and mutant muscle in (A). In longitundinal sections, plectin and desmin colocalized at Z-disks with no detectable difference in the staining patterns of *Plec* ^+/+^ and mutant tissues. (C) Cardiac muscle immunolabeled for plectin. Note similar staining pattern of plectin at Z-disks and intercalated disk structures (arrowheads) in all tissues. Bar in C, 50 µm (representative for A–C). (D–F) Sarcomeric units in soleus (D) and cardiac muscle (E,F) labeled with anti-α-actinin (D,E) and anti-desmoplakin (F) antibodies. Note normal alignment of sarcomers in both skeletal and heart muscle (D,E) as well as well preserved intercalated disk structures in cardiac muscle of mutant mice (F). Bar in F, 50 µm (representative for D–F).

When immunolabeled frozen sections of skeletal and heart muscle tissues of adult mutant mice were compared with those from wild-type mice no noticeable differences in plectin's staining patterns or signal intensities were observed. Plectin expression at Z-disks of mutant mice was well preserved in both types of muscle, and no signs of disorganization of the contractile apparatus or of intercalated disks were apparent (Figure 4A–4F). Likewise, no differences in plectin expression levels were observed by immunoblotting (Figure S3).

### Cultured Ogna keratinocytes show impaired formation of HD-like protein complexes, reduced osmotic shock resistance, and increased migration potential

**Figure 5:**
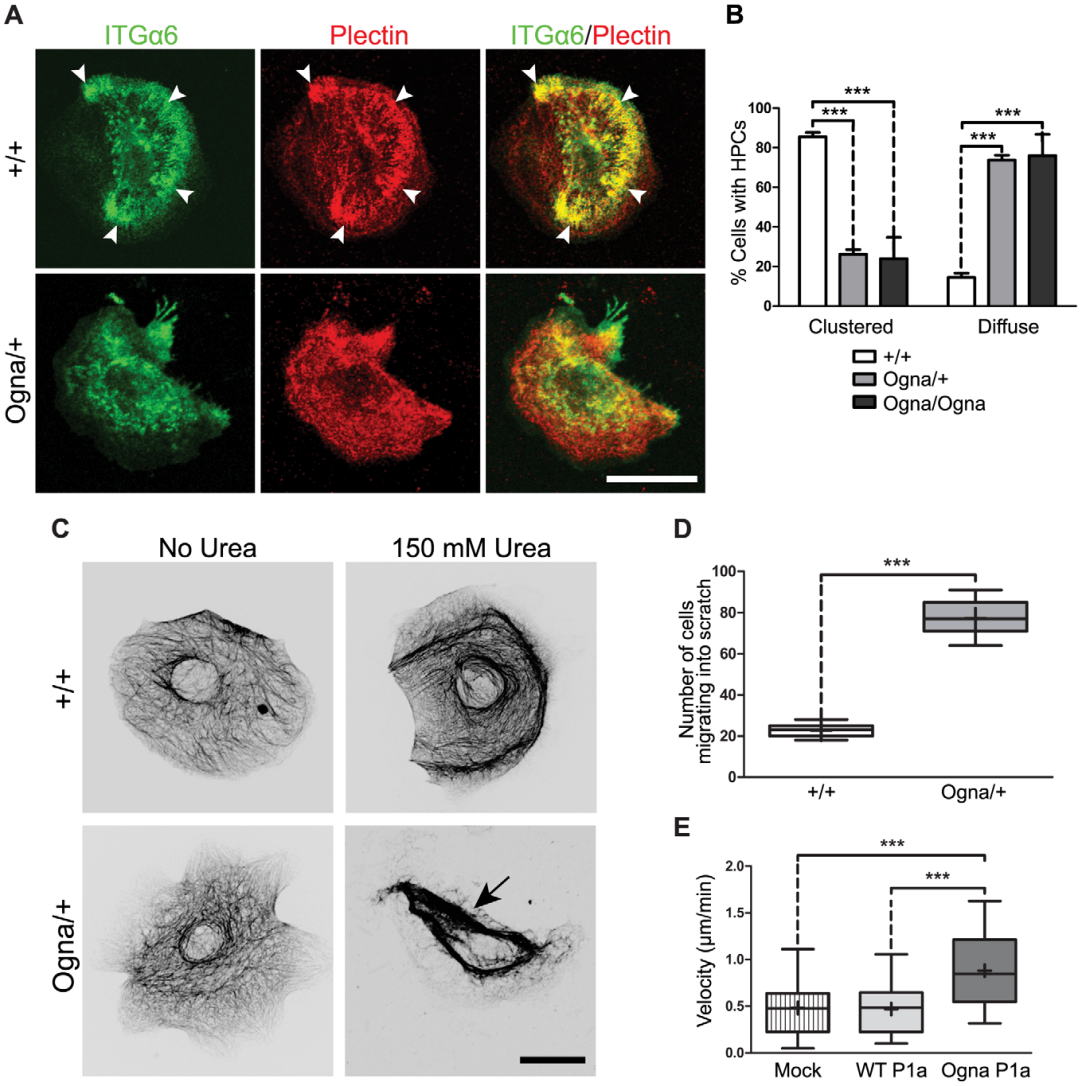
Compromised ex vivo formation and function of HPCs in Ogna keratinocytes. (A) Immunolocalization (double labeling) of ITGα6 and plectin in primary keratinocytes isolated from newborn mice. In wild-type (+/+) keratinocytes ITGα6 and plectin show codistribution in densely clustered HPCs (arrowheads) contrasting the more diffuse distribution in Ogna keratinocytes. Bar, 20 µm. (B) Column diagram showing proportions (%) of wild-type (+/+) and mutant keratinocytes having formed (clustered), or lacking (diffuse) HPCs. Data are shown as mean values from cell counts (>100/genotype) in randomly chosen optical fields from three independent experiments ±95% CI. *** P<0.001, two-way ANOVA with Bonferroni post test. (C) Immunofluorescence microscopy images (contrast–enhanced by conversion to grey scale and inversion of contrast) of keratin 5 filament networks in hyperosmotic shock-treated (+urea) and non-treated keratinocytes. Note more advanced network collapse in *Plec* ^Ogna/+^ (arrow) compared to wild-type keratinocytes. Bar, 20 µm. (D) Comparison of cell numbers re-populating scratch wounds (n = 15) 16 hours after wound infliction. Box and whisker plots as in Figure 2. *** P<0.001, unpaired two-tailed t-test. (E) Migration velocities of keratinocytes overexpressing wild-type and Ogna-P1a. Immortalized *Plec* ^+/+^ keratinocytes, transfected with either full-length wild-type (WT) P1a (n = 30 cells), or Ogna P1a (n = 34 cells), or empty (GFP-expressing) vector (mock, n = 30 cells), were monitored for migration over a period of 20 hours. Box and whisker plots as in Figure 2). *** P<0.001, one-way ANOVA with Tukey post test for multiple comparisons.

Cultured keratinocytes form dynamic HD-like protein complexes (HPCs [22], [23]), whose assembly is dependent on a functional laminin322–ITGα6β4–plectin–K5/K14 backbone [6], [10], [22]–[24]. To assess HPC formation in Ogna versus wild-type cells, we isolated primary keratinocytes from newborn mice and cultured them at low cell density in keratinocyte growth medium (KGM) supplemented with 0.3 mM Ca^2+^ (KGM/0.3) to stimulate HPC formation [10], [23]. In *Plec* ^+/+^ keratinocytes, ITGα6 and plectin were found to colocalize in typical bow-like patches at the substratum-attached surface of cells (Figure 5A, arrowheads), whereas in *Plec* ^Ogna/+^ cells, plectin showed a diffuse cytoplasmic distribution and ITGα6 clustering at the basal cell surface was greatly reduced (Figure 5A). Similar alterations were observed in *Plec* ^Ogna/Ogna^ keratinocytes (data not shown). A quantitation revealed that >80% of wild-type keratinocytes had formed ITGα6/plectin-positive HPCs, with the rest (<20%) showing a diffuse distribution (Figure 5B). In *Plec* ^Ogna/+^ and *Plec* ^Ogna/Ogna^ keratinocytes, the situation was reversed (Figure 5B). Compromised HPC formation was also observed in cluster-forming *Plec* ^Ogna/+^ and *Plec* ^Ogna/Ogna^ keratinocytes grown at higher cell densities (Figure S4A, and data not shown). At the transition from subconfluence to post-confluence, when keratinocytes began to stratify, fewer HPCs where detectable in *Plec* ^+/+^ keratinocytes (see also [25]), whereas hardly any HPCs could be observed in *Plec* ^Ogna/+^ and *Plec* ^Ogna/Ogna^ keratinocytes (Figure S4B, and data not shown).

Regarding K5/K14 IF network organization no differences between *Plec* ^+/+^ and mutant keratinocytes were apparent (Figure 5C). In contrast to plectin-null keratinocytes which exhibit keratin IF networks of enlarged mesh size along with increased keratin IF-bundling [26], the keratin IF networks in *Plec* ^Ogna/+^ keratinocytes displayed mesh sizes similar to that of *Plec* ^+/+^ cells, with no signs of filament-bundling. Likewise, full-length versions of Ogna- and wild-type P1a, when ectopically expressed in plectin-null keratinocytes, showed similar potentials for rescuing their aberrant keratin network cytoarchitecture (Figure S5).

Hypo-osmotic shock, a common physiological stress that causes a transient reorganization of the cytoskeleton [27], can be used to assess the stability of keratin IF anchorage at HPCs [26]. When low cell density cultures of primary keratinocytes isolated from newborn wild-type animals were exposed to 150 mM urea, in accordance with previous studies [26], the keratin IF network appeared more bundled, but otherwise intact (Figure 5C). In contrast, under similar conditions, the keratin IF network of *Plec* ^Ogna/+^ keratinocytes had retracted from the cell periphery, showing prominent lateral bundling in central parts of the cytoplasm, sometimes followed by a complete lateral collapse in form of massive filamentous bundles (Figure 5C, arrow). As documented in Figure S6, ∼70% of *Plec* ^Ogna/+^, but only ∼13% of *Plec* ^+/+^ urea-exposed keratinocytes showed keratin IF network collapse.

When keratinocyte monolayers were subjected to scratch wound closure assays to assess whether impaired HPC formation in Ogna cells was reflected in an increased migratory potential of cells, *Plec* ^Ogna/+^ keratinocytes were found to repopulate the wound area significantly faster than *Plec* ^+/+^ cells (Figure 5D). To confirm this in a single cell-targeted approach, we transiently transfected immortalized *Plec* ^+/+^ keratinocytes with expression plasmids encoding GFP fusion proteins of full-length P1a with or without the mutation, and monitored migration of transfected cells by time-lapse video microscopy. Whereas *Plec* ^+/+^ keratinocytes transfected with wild-type P1a exhibited an average migration speed of 0.47 µm/min, very similar to that of mock-transfected control cells (0.48 µm/min), the average migration velocity of cells transfected with Ogna-P1a was 0.88 µm/min, and thus significantly higher (Figure 5E, and Video S1 and Video S2). This indicated a dominant-negative effect of the mutation on HPC stability leading to faster cell migration.

### The Ogna mutation decreases the stability of the plectin rod

**Figure 6:**
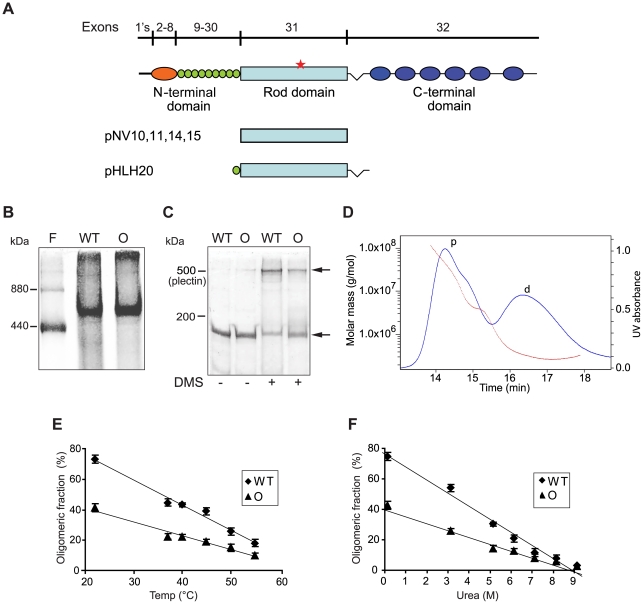
Oligomerization of plectin's RD. (A) Schematic representation of plectin's domain structure with exon allocation of subdomains and recombinant proteins expressed in baculovirus. 1's stands for 11 first exons alternatively spliced into exon 2. Highlighted are plectin's actin-binding domain (orange), the 9 spectrin repeats (green), the α-helical rod domain (light blue), and the C-terminal domain comprising 6 plectin repeats (dark blue). Red star, position of the Ogna mutation. pNV10 and pNV11, recombinant His-tagged wild-type and Ogna plectin RDs (135 kDa); pNV14 and pNV15, corresponding GST-tagged versions (158 kDa); pHLH20/wt and pHLH20/Ogna, recombinant His-tagged wild-type and Ogna versions of the RD flanked by the 9^th^ spectrin repeat and the linker region between plectin's rod and the C-terminal region (170 kDa). (B) BN-(4–10%)-PAGE of recombinant wild-type (WT) and Ogna (O)-RDs using ferritin (440 and 880 kDa) as size marker. (C) Chemical cross-linking of RDs. Purified RD samples before and after cross-linking with DMS were analyzed by SDS-5%-PAGE. Note that i) the major cross-linked species (upper arrow) migrated just above the ∼500 kDa size marker (plectin), and ii) a fraction of both proteins could not be cross-linked in solution and was detected as monomers (lower arrow). (D) Molecular mass measurement by SEC-MALS analysis. The blue line traces the absorbance at 280 nm of the eluate from a Superose 6 10/300GL column as a function of time. The red dotted line represents the weight-average molecular weight of the species in the eluate, calculated from refractive index and light-scattering measurements. The protein peaks containing RD dimers and high molecular mass polymers are labeled with d and p, respectively. (E,F) Dissociation of plectin RD oligomers as a function of temperature and urea concentration. Samples were preincubated at increasing temperatures or concentrations of urea, cross-linked with DMS, and resolved by SDS-PAGE. The relative percentages of oligomers in each sample, determined by densitometric analysis of the gel lanes, were plotted as a function of temperature (E) or added urea (F). Data are shown as mean ±SD of three independent experiments performed in duplicates. The solid lines show the linear regression fit of the data (r^2^≥0.9689).

To examine whether the Ogna-mutation has a negative impact on plectin dimerization or oligomerization, purified recombinant His-tagged wild-type and mutant versions of plectin's RD (Figure 6A; M_r_ of monomers: 135K) expressed in Sf9 cells were subjected to blue native gel electrophoresis (BN-PAGE), gel filtration, and chemical cross-linking. In BN-PAGE, both plectin RD versions migrated as single major molecular species with apparent molecular masses of ∼500 kDa (as estimated from their position between the 880 kDa and 440 kDa ferritin protein bands run as reference). This was consistent with plectin RDs forming tetramers (expected molecular mass ∼540 kDa) in solution. Additionally observed >880 kDa species, hardly entering the gels, likely corresponded to oligomers of higher order (Figure 6B). Distinct bands with apparent lower molecular masses corresponding to putative dimers and monomers were never detected, despite the application of several different electrophoresis conditions (data not shown). The analysis of RDs covalently cross-linked using DMS (a homobifunctional agent that reacts with primary amines), followed by SDS-PAGE, revealed a predominant cross-linked product migrating above the position of full-length plectin (∼500 kDa), similar to the single major molecular species observed in BN-PAGE (Figure 6C). In addition, a fraction of both cross-linked proteins, likely representing high molecular mass aggregates, just entered the stacking gels but did not get electrophoreticaly separated (data not shown). No assembly intermediates were detected when the cross-linking reaction was carried out under sub-stoichiometric conditions (low concentrations of DMS, data not shown). To more accurately determine the molecular masses of RD oligomers we performed size exclusion chromatography with on-line multiangle laser light scattering (SEC-MALS), a technology providing a reliable, direct measurement of the absolute molecular masses of proteins independent of their shape and elution volume [28], [29]. SEC-MALS chromatograms of wild-type RDs yielded two major protein peaks, the one eluting first corresponded to a mixture of protein aggregates of molecular masses ranging from 1,000 to >100,000 kDa (Figure 6D), the second to a single protein species with a defined molecular mass of 267 kDa (Figure 6D). Since the calculated molecular mass for the monomeric RD of plectin is 135 kDa, the protein eluting in the second peak must have been a dimer (expected molecular mass = 270 kDa). Unexpectedly, a distinct protein peak corresponding to a tetrameric form could not be detected. Based on these results we concluded that the putative tetramers observed in BN-PAGE and in SDS-PAGE after chemical cross-linking were in fact dimers showing an aberrant migration behavior due to their extended shape. This conclusion was supported by the fact that the elution of the dimeric RD species from the SEC column was significantly faster than that of a globular protein of similar molecular mass (data not shown). The protein peak containing the RD aggregates displayed a trailing shoulder containing proteins of molecular masses between 3,500 and 5,000 kDa (Figure 6D), probably representing a small population of energetically stable RD polymers. Three SEC-MALS experiments using different protein purification protocols and sample solvents resulted in practically identical molecular mass determinations. Summarized, our data suggested that in its native state the RD is a dimer that can self-associate into higher-order oligomers.

To compare their stability, oligomers of wild-type and mutant RDs formed in solution were exposed to increasing temperatures and concentrations of urea, prior to chemical cross-linking and analysis by SDS-PAGE. Densitometric scanning of gels revealed that the RD fraction found in the dimeric form, even without denaturant was always higher for wild-type (∼75%) compared to Ogna (∼40%) (Figure 6E and 6F). This trend was retained as dimers dissociated into monomers with rising temperature (Figure 6E) or concentrations of urea (Figure 6F). Midpoints calculated on the basis of their apparent linear dissociation kinetics were 44°C and 4.45 M urea for the wild-type RD, compared to 41°C and 4.21 M urea for the Ogna RD. These results suggested that the Ogna mutation had a minor destabilizing effect on the RD dimer.

**Figure 7:**
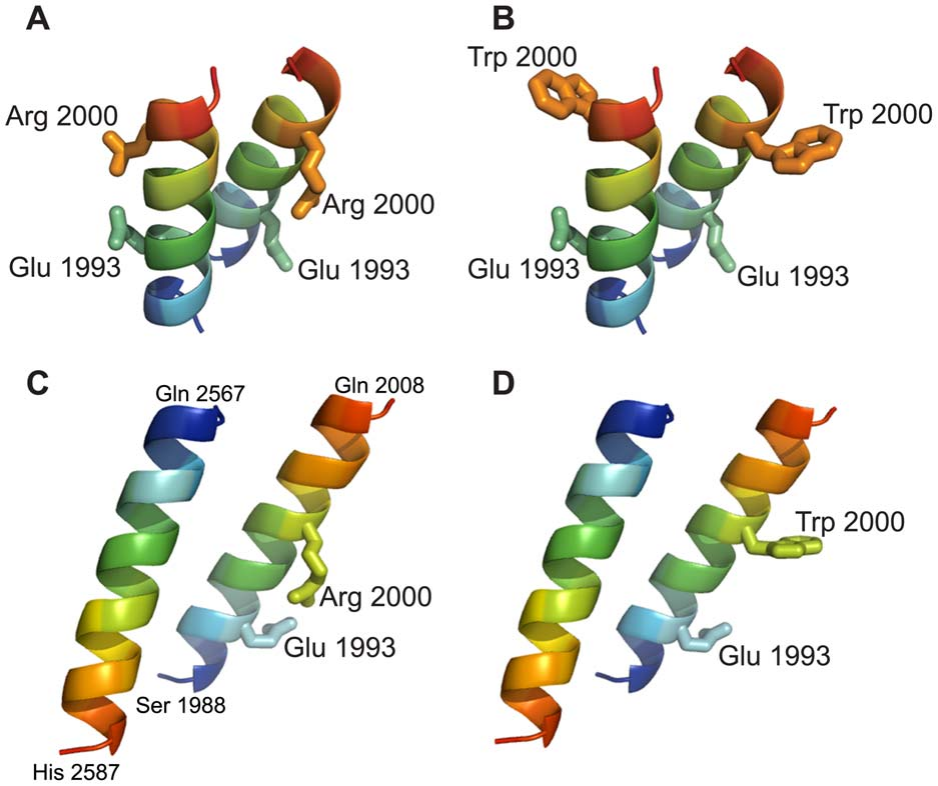
Molecular modeling of RD fragments harboring the p.Arg2000Trp mutation. Ribbon views of three-dimensional models of parallel (A,B) and antiparallel (C,D) fragments of wild-type coiled-coil dimers (A and C), and of their p.Arg2000Trp mutant versions (B and D). Chains are colored according to blue-to-red (N to C terminus) scheme. The parallel dimeric coiled-coil model contains two copies of the segment 1988–2003 and the antiparallel dimeric model contains the segments 1988–2008 and 2587–2567. Note that arginine 2000 can form an intrahelical salt bridge with glutamine 1993, which is disrupted by the p.Arg2000Trp mutation.

A small moiety of the long coiled-coil RD of human plectin harboring the p.Arg2000Trp Ogna mutation was modeled in silico. In a dimeric parallel as well as in a dimeric antiparallel model of the wild-type version an intra-chain salt bridge was observed in the native protein between residues Arg 2000 and Glu 1993 (Figure 7A and 7C). The three-dimensional models of the corresponding p.Arg2000Trp mutant version suggested that its thermodynamic stability was lower than that of the wild-type protein. In both orientations the p.Arg2000Trp mutation abolished the electrostatic interaction with Glu 1993 and concomitantly exposed the hydrophobic side-chain of tryptophan to the solvent (Figure 7B and 7D). This situation is energetically unfavorable and it can be hypothesized that the Trp 2000 side-chain tends to enter into the apolar inter-helical interface, leading to a local unfolding of the helix and disruption of the coiled-coil arrangement. This hypothesis is supported by coiled-coil tendency predictions, based on the amino acid sequence. Three methods used (COILS, Paircoils2, and Multicoil) suggested a decrease of the coiled-coil tendency around amino acid position 2000 upon substitution of arginine by tryptophan. For the rest of the RD, the tendency to form a coiled-coil is unaffected by the Ogna mutation. Thus, the prediction by the computational model that the Ogna mutation causes a local disruption of the coiled-coil, decreasing its stability, is fully compatible with our biochemical findings.

To assess whether wild-type and Ogna RDs could form heterodimers, we co-infected Sf9 cells with baculoviruses expressing His-tagged wild-type and GST-tagged Ogna RDs (and vice versa). By pulling down the oligomeric products via one of the tags, hetero-oligomer formation could indeed be demonstrated (Figure S7A and S7B, and data not shown).

### Formation of filamentous polymers through lateral association of plectin's RD

**Figure 8:**
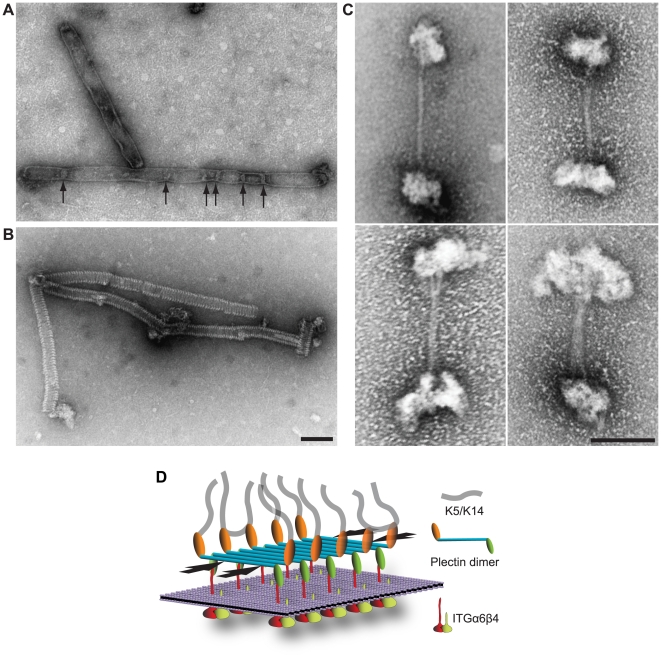
RD polymer formation and model highlighting lateral RD association as a novel HD-stabilizing force. (A,B) Electron microscopy of polymeric RD structures. pHLH20/wt-encoded recombinant versions of plectin's RD (Figure 6A) were incubated in 50 mM sodium phosphate, pH 7.4, 300 mM NaCl, 170 mM imidazole, and 1 mM PMSF for 1 hour at 37°C, before being processed for uranyl acetate staining and electron microscopy. Note that the slightly larger diameter of flat sheet/envelope-like structures visualized in (A) would correspond to that expected for collapsed (flat) tube-like structures visualized in (B). Constrictions (A, arrows) of sheet-like structures may represent transition states between flat (collapsed) and tube-like structures. Bar, 100 nm. (C) Electron microscopy of oligomeric dumbbell-like structures assembled from intact (full-length) plectin molecules. Plectin samples purified from rat glioma C6 cells in 7 M urea were dialysed into 2 mM Tris-HCl, pH 8.0, 5 mM β-mercaptoethanol, and 3 mM PMSF at 4°C overnight. Samples were then incubated in the presence of 10 mM CaCl_2_ at 37°C for 1 hour, and thereafter processed for negative staining electron microscopy. Note correlation between increasing sizes of globular end domains and stalk diameters of the four specimens shown. Bar, 100 nm. (D) Schematic model depicting the major protein-protein interactions that provide the mechanical stability of HDs. Interaction of P1a dimers with ITGβ4 and K5/K14 provides a vertical force component, whereas the ITGβ4-induced lateral association of multiple dimeric P1a molecules (in an anti-parallel fashion via their RDs) could generate an additional horizontal force component (arrows), parallel to the plasma membrane (violet sheet). Note that individual proteins are not drawn to scale and BPAG1e and BPAG2 are not depicted. In contrast to classical models of HD protein organization, the sheet-like association of plectin molecules in our model is better suited to incorporate the dimensions of plectin molecules (>230 nm) within the 50–60 nm thick HD inner plate.

BN-PAGE, chemical cross-linking, and SEC-MALS, all provided evidence for the aggregation of plectin's RD into high molecular mass structures that were out of the resolution range of these methods. To investigate whether this material represented ordered structures, recombinant protein fragments corresponding to the α-helical RD, or to the RD with flanking N- and C-terminal plectin sequences (see Figure 6A), were subjected to negative-staining electron microscopy. All samples tested formed higher-order structures, most notably long filamentous polymers (Figure 8A and 8B; and data not shown), that were stable at 4°C for up to several months and could only be disassembled by 7 M urea. The most striking assemblies observed resembled either flat, elongated sheets or envelopes having a width of 45–52 nm with some of them displaying periodic constraints (∼35 nm) (Figure 8A, arrows), or highly ordered (paracrystalline) cylindrical structures of uniform diameter (∼36 nm) (Figure 8B), both of variable length. In addition, we observed networks of smooth and partially branched filaments (with diameters varying from 17 to >70 nm) and filaments with more regular diameters (∼13 nm) displaying a tangled topology reminiscent of IFs (data not shown). While the latter structures apparently were assembled by lateral alignment of α-helical RDs in a staggered, partially overlapping fashion, the cylindrical and probably also the sheet-like structures, most likely were assembled from stacks of disk- or screw-like, RD subunits which laterally associated with each other to form a helix. Ogna RDs formed similar structures (Figure S7C–S7E), indicating that they, too, were capable of lateral self-association. However, a semi-quantitative analysis revealed a trend towards shorter sheet/cylinder-like polymeric RD structures in case of the Ogna mutant, compared to wild-type (Figure S7F–S7H). Polymeric rod structures were largely resistant to changes in the ionic strength of the solvent, suggesting that ionic interactions contributed little to filament stability. To assess whether lateral association of plectin's RD could also be observed with full-length plectin molecules, native plectin was purified from cultures of rat glioma C6 cells [30] and subjected to negative-staining electron microscopy. Among a series of different conditions tested, including those leading to polymer formation of recombinant RDs, solubilization of glioma C6 cell plectin in low ionic strength buffer in the presence of 10 mM CaCl_2_ was found to be favorable for oligomerization of plectin. The predominant ordered structures observed under these conditions resembled dumbbells, reminiscent of the shape characteristic for plectin dimers, as previously visualized using the rotary shadowing technique [30]. As exemplified in Figure 8C, most of these structures were >200 nm in length, with globular end domains of irregular sizes and RDs varying in diameters from <4 nm to >18 nm. Similar structures could be observed also with full-length plectin purified from an epithelial (804G) cell line (data not shown). As the rods of dimeric plectin molecules measure ∼2 nm in diameter [30], we suggest that the structures shown in Figure 8C represent oligomers of plectin dimers formed via lateral, multi-layered association of their RDs. The lateral self-association of plectin molecules leading to stable polymeric structures could play an important role for HD integrity and inner plate formation (Figure 8D; and text below).

### Plectin expression in Ogna epidermis is reduced below a critical level necessary for tissue integrity

**Figure 9:**
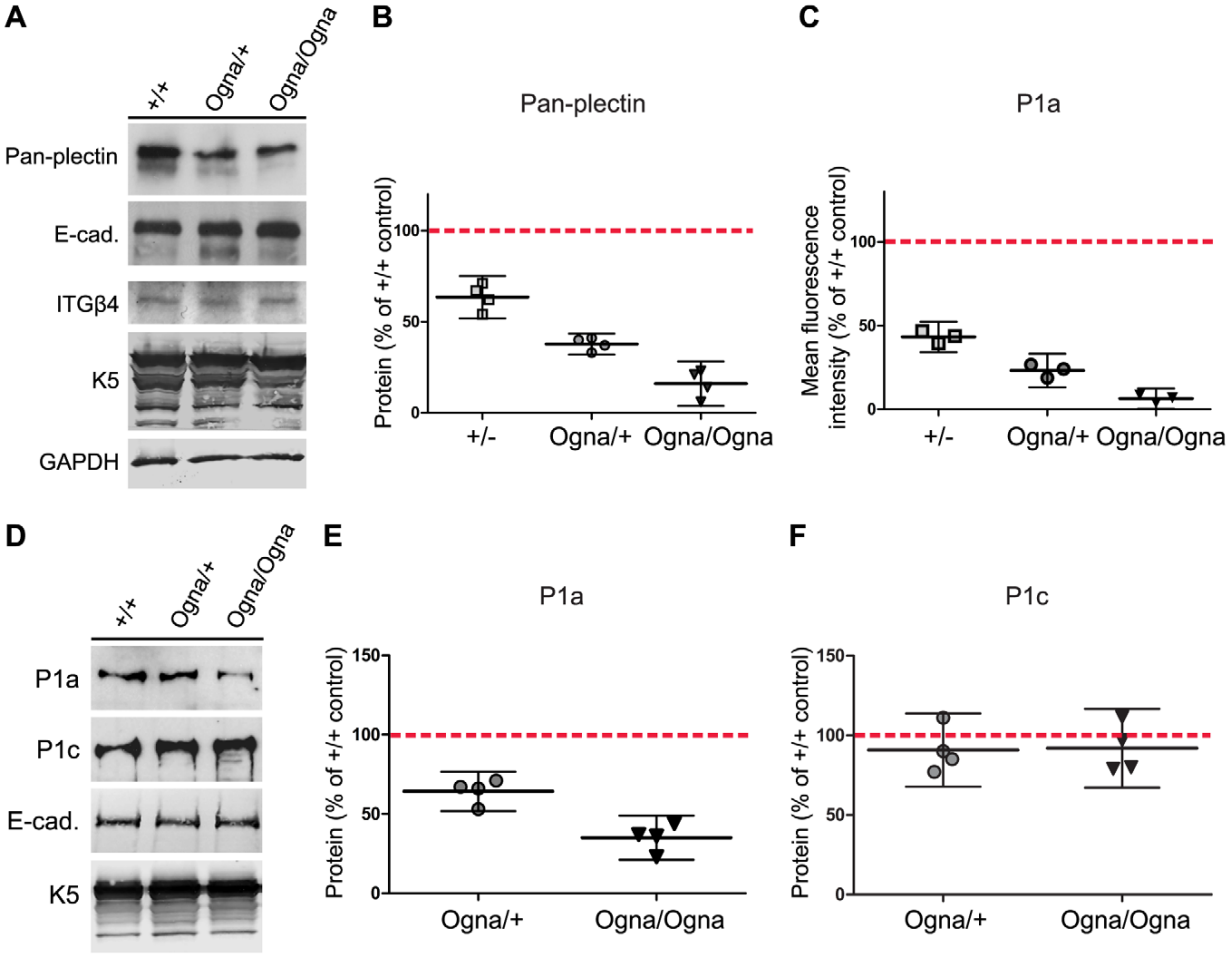
Downregulation of P1a protein levels in Ogna keratinocytes. (A) Immunoblotting of extracts prepared from *Plec* ^+/+^, *Plec* ^Ogna/+^, and *Plec* ^Ogna/Ogna^ epidermis using antibodies to proteins indicated. Sample loading was normalized to equal total protein contents and verified by immunoblotting using antibodies to GAPDH. (B) Densitometric quantification of plectin protein levels in mutant epidermal cell extracts relative to that in *Plec* ^+/+^ samples (100%, red broken line) using E-cadherin as loading control. Data are shown as scatter dot plot. Values represent independent experiments (n = 4), horizontal lines indicate the mean, and error bars show 95% CI. Statistical significance between all genotypes was demonstrated by one-way ANOVA with Tukey posttest for multiple comparisons (P<0.01). Note that data shown for heterozygous plectin-null mice (+/−) were obtained in independent experiments (not shown in A). (C) Semiquantitative confocal microscopy analysis (n = 3 per group) of P1a protein levels in mutant relative to *Plec^+/+^* epidermis (100%, red broken line), using identical image settings. Data presentation as in (B). Statistical significance between all genotypes (P<0.001) was demonstrated by one-way ANOVA with Tukey posttest for multiple comparisons. (D) Immunoblotting of cell lysates prepared from confluent primary keratinocytes grown in KGM/0.3 using antibodies to proteins indicated. Note, samples contained equal amounts of K5 and E-cadherin. (E,F) Densitometric quantification of plectin isoform levels in primary mutant keratinocytes relative to that in *Plec* ^+/+^ samples (100%, red broken line) using K5 as loading control. Data presentation as in (B). In (E), statistical significance between all genotypes (P<0.05) was demonstrated by one-way ANOVA with Tukey posttest for multiple comparisons.

To investigate the mechanism leading to a reduction of HDs in Ogna epidermis, we quantified the expression levels of major HD proteins by immunoblotting. In epidermal protein extracts from adult Ogna mice we found plectin to be reduced to levels below that of mice heterozygous for the plectin-null allele (*Plec^+/−^*), whereas the levels of ITGβ4 and K5 were not significantly altered (Figure 9A and 9B). Using isoform-specific antibodies we were unable to evaluate the relative expression levels of different plectin isoforms in epidermal protein extracts by immunoblotting, because the epitopes in the very N-terminal domain of plectin apparently were lost during the vigorous and lengthy protein extraction procedure. Consequently we resorted to comparing the intensity of P1a-specific immunofluorescence signals in wild-type and mutant epidermis by confocal microscopy [31]. This type of analysis revealed that in *Plec^Ogna/+^* and *Plec^Ogna/Ogna^* epidermis the protein level of P1a at the basal cell membrane of basal keratinocytes was reduced to ∼25% and ∼6%, respectively, compared to wild-type (Figure 9C). In contrast, P1a levels in the epidermis of heterozygous *Plec^+/−^* mice were reduced to only ∼48% (Figure 9C). Since *Plec^+/−^* mice do not display any skin pathology [9], [17], the amount of plectin expressed in their epidermis defines the threshold for plectin expression necessary for this type of tissue to withstand mechanical stress. Consequently, our results suggested that the epidermal P1a levels in Ogna mutant mice were too low to support formation of HDs in sufficiently high numbers. Furthermore, they indicate that the mutant protein adversely affected the wild-type protein, as is typical for dominant negative mutations. Protein expression levels of plectin isoforms were also measured by immunoblotting of primary keratinocyte cell lysates. In lysates from *Plec^Ogna/+^* and *Plec^Ogna/Ogna^* keratinocytes P1a protein levels were found to be reduced to ∼60% and ∼35% (Figure 9D and 9E), respectively, compared to *Plec^+/+^* cells; in contrast, the protein levels of P1c remained normal under these conditions (Figure 9D and 9F).

Quantitative real-time PCR analysis of RNA isolated from mutant mouse epidermis or primary keratinocyte cultures revealed hardly any changes in transcript levels of total plectin, P1a, or P1c, relative to wild-type samples (Figure S8A and S8B; and data not shown). Thus, we explored next whether in the epidermis P1a was subjected to selective proteolytic degradation.

### The Ogna mutation predisposes plectin's rod to degradation by epidermis/keratinocyte-specific proteases

**Figure 10:**
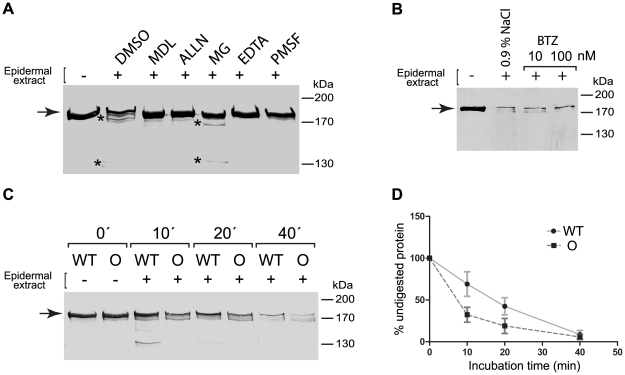
The Ogna mutation sensitizes plectin's RD to degradation by epidermis-specific proteolytic activities. (A) Aliquots (10 µg) of GST-tagged wild-type RD (Figure 6A) were incubated (30 min, 30°C) with (+) or without (−) epidermal protein extract (5 µg) in the presence of the protease inhibitors indicated [MDL, MDL-28170 (50 µM); ALLN, N-Acetyl-Leu-Leu-Nle-CHO (5 µM); MG, MG132 (Z-Leu-Leu-Leu-al, 20 µM); EDTA (10 mM), PMSF (2 mM)], or vehicle (DMSO) alone. Samples were separated by SDS-8% PAGE and RDs were detected by immunoblotting using anti-plectin mAb 10F6. Note that RDs exhibit electrophoretic mobilities slightly lower than expected for a protein with a calculated molecular mass of ∼158 kDa. Also note multiple RD degradation products (*) in lanes DMSO and MG. (B) Assay conditions as in (A), except that 10 or 100 nM Bortezomib (BTZ), and 0.9% (w/v) NaCl were used as protease inhibitor and sole vehicle, respectively. (C) Degradation of wild-type (WT) and mutant plectin RDs (O) by epidermal protease(s) as a function of incubation time. Assay conditions as in (A), except that no protease inhibitors were added and incubations times varied as indicated. Note faster degradation of the Ogna compared to the wild-type RD version. Arrows in (A–C) denote the position of intact RD proteins. Note faster degradation of the Ogna RD compared to the wild-type rod version. (D) Degradation kinetics of wild-type versus Ogna RD. The relative amounts of intact RD (as determined by densitometry) were plotted as a function of incubation time with epidermal protein extract. Data are shown as mean ±SEM of three independent experiments.

As plectin is cleavable in its RD by various protease activities, including caspases [32], [33] and calpain-1 [34], we set up an in vitro assay to assess epidermal cell extracts for RD-degrading activities. We found that the recombinant version of plectin's RD was almost completely degraded within 60 min when subjected to this assay. To identify the proteolytic activities targeting plectin, degradation assays were performed in the presence of various protease inhibitors. As shown in Figure 10A, degradation was inhibited by EDTA, indicating the involvement of a divalent ion-dependent protease. In fact, two inhibitors of the Ca^2+^-dependent protease calpain, MDL-28170 and ALLN, efficiently blocked degradation. In addition, MG132, a proteasome inhibitor that additionally inhibits calpains [35], was able to partially inhibit degradation. No inhibition was detected using the proteasome-specific inhibitor Bortezomib [36], [37] (Figure 10B). Specific cleavage of plectin's RD, as well as of native full-length plectin (purified from cultured C6 cells [30]), was observed also when purified samples of calpain-1, instead of crude cell extracts, were used (Figure S9A and S9B). However, in this case multiple cleavage products were generated from both samples without complete protein degradation, suggesting additional proteolytic activities with the potential to degrade plectin to be present in epidermal cell extracts. Inhibition of epidermal proteolytic activities was also observed using PMSF, an inhibitor of serine proteases (Figure 10A), whereas inhibitors of aspartic proteases, such as pepstatin A had no effect (data not shown).

A zymographic analysis confirmed the presence of multiple gelatinolytic serine proteases in epidermal protein extracts (Figure S9C). Moreover, using similar assays we found protein extracts of wild-type and mutant epidermis to contain comparable amounts of serine as well as of calpain protease activities (data not shown). These data suggested that plectin can be efficiently degraded by calpains and serine proteases present in the epidermis. Importantly, no degradation of plectin's RD was observed when crude cell extracts from skeletal muscle instead of epidermis were used (data not shown). Comparing the degradation kinetics of recombinant wild-type and Ogna RDs exposed to epidermal protein extracts, we found that the RD version containing the Ogna mutation was degraded faster than the one without it (Figure 10C and 10D). This suggested that the mutation sensitized the RD to proteolytic activities specifically occurring or activated in keratinocytes. Mass spectrometry analysis of proteins pulled down from keratinocyte extracts using plectin RDs (wild-type and Ogna) as baits did not identify any proteins showing differential binding (results not shown).

### Abundant expression of activated calpain-1 in mouse epidermis

Focusing on calpain-1 as a candidate protease involved in the degradation of Ogna P1a, we first assessed whether the protease showed colocalization with P1a on frozen skin tissue sections. Using antibodies recognizing the large subunit of calpain-1, strong signals were observed in the granular and basal cell layers of the epidermis (Figure S10A). Notably, in the majority of basal keratinocytes, calpain-1 showed colocalization with ITGβ4 at their basal cell membrane (Figure S10A), suggesting its close spatial relationship also with P1a. Activation of calpain-1 is universally accompanied by autoproteolytic cleavage of its inactive full-length form (80 kDa) into an activated 76 kDa form, as shown both in vitro and in vivo [38]–[42]. To determine whether calpain-1 expressed in mouse epidermis was enzymatically active, we monitored its autoproteolytic cleavage using immunoblotting. We found that the predominant fraction of calpain-1 contained in epidermal cell lysates corresponded to the autoproteolytically cleaved form (Figure S10B), indicating that the epidermis contains high levels of calpain activity. When epidermal tissue extracts were subjected to casein zymography [43], similar results were obtained (data not shown). These observations suggested that in normal mouse epidermis, most of calpain-1 is in an active form.

### Calpain activation in cultured keratinocytes causes degradation of hemidesmosomal proteins

In contrast to mouse epidermis, primary and immortalized *Plec^+/+^* keratinocytes cultured ex vivo contained hardly any autoproteolytically cleaved calpain-1 (Figure S10B and Figure S11A). To optimally activate calpains, we treated immortalized *Plec^+/+^* keratinocytes with the Ca^2+^ ionophore ionomycin in the presence of high concentrations (1.8 mM) of extracellular Ca^2+^ [44] and determined whether hemidesmosomal proteins were degraded under these conditions. Treatment of keratinocytes with 5 µM ionomycin for up to 5 hours led to a strong activation of calpain-1 and a gradual decrease in P1a and ITGβ4 protein levels (Figure S11A–S11C), supporting the notion that their degradation was mediated by calpains. Accordingly, the calpain inhibitor MDL-28170 blocked the ionomycin-induced degradation of P1a and ITGβ4 (Figure S11A–S11C). The kinetics of P1a degradation were slower compared to that of ITGβ4, suggesting that P1a was more resistant to calpain-mediated degradation than ITGβ4. In accordance with the reduction of ITGβ4 and P1a expression levels during ionomycin treatment, we observed a progressive loss of HPCs using confocal immunofluorescence microscopy (Figure S11D). These results strongly suggested that activated calpain-1 targets and degrades HPC-associated ITGβ4 and P1a.

### Partial rescue of P1a degradation in Ogna keratinocytes through inhibition of calpains ex vivo and in vivo

**Figure 11:**
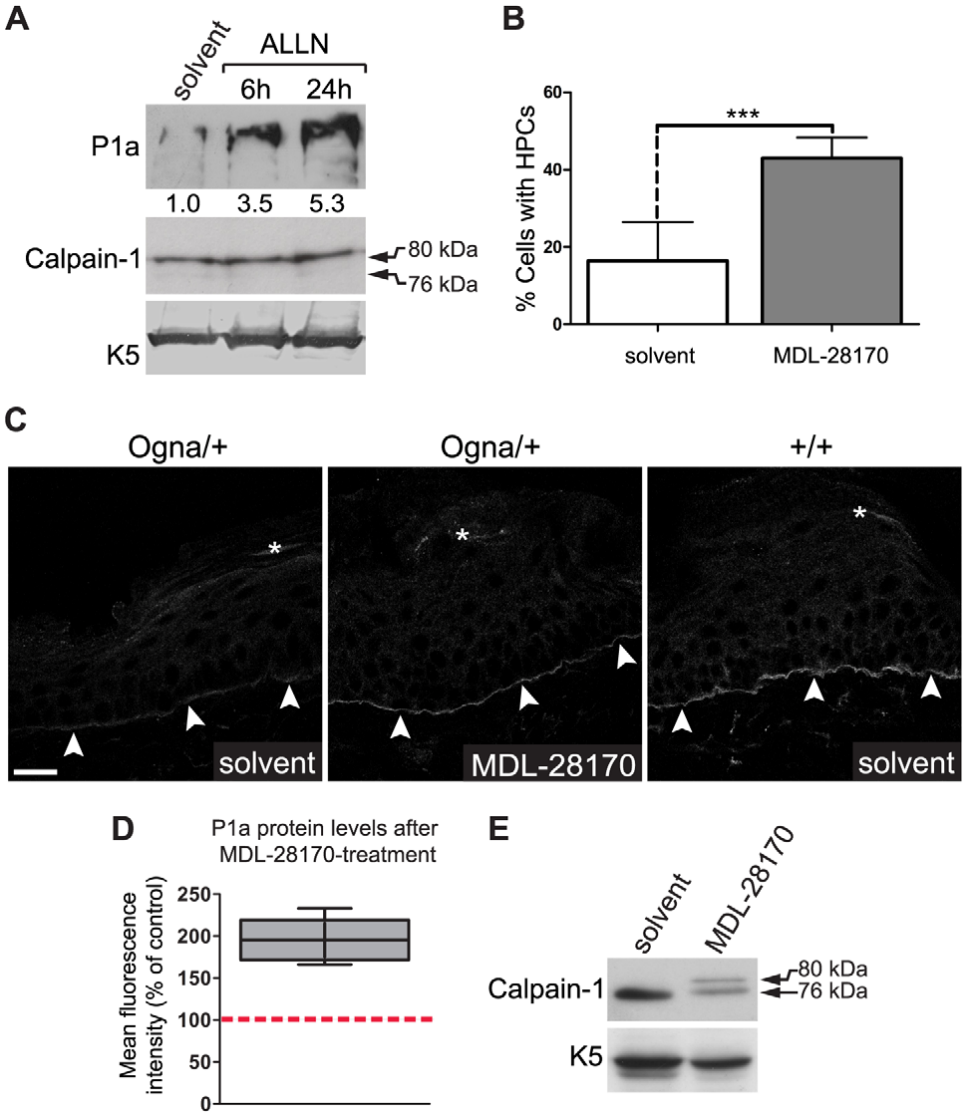
Calpain inhibitors restore P1a expression and HD formation in Ogna keratinocytes and Ogna mice. (A) Immunoblotting of cell lysates from primary *Plec^Ogna/Ogna^* keratinocytes grown to ∼70% confluence in KGM/0.3, and exposed to either DMSO (solvent) alone, or calpain inhibitor ALLN (10 µM) for up to 24 hours. Note, samples analyzed contained equal amounts of calpain-1 and K5. Numbers below lanes represent protein ratios relative to an arbitrary level of 1.0 assigned to the control (solvent) sample. One of three experiments is shown. (B) Primary *Plec^Ogna/Ogna^* keratinocytes grown as in (A) were exposed to DMSO (solvent) alone, or calpain inhibitor MDL-28170 (50 µM) for 48 hours. The bar diagram shows proportions (%) of solvent- and MDL-28170-treated cells having formed HPCs. Data shown represent mean values of cell counts (>500/genotype) in randomly chosen optical fields from three independent experiments ±95% CI. *** P<0.001, unpaired Student's t-test. (C) Visualization of P1a on frozen sections of mouse tail skin using P1a-specific antibodies. Note that in tail skin of *Plec^Ogna/+^* mice treated with MDL-28170 there was a clear increase in P1a levels compared to mice treated with DMSO (solvent) only; even though the restored P1a levels did not reach the levels of *Plec^+/+^* controls. Arrowheads, basal cell membrane of basal keratinocytes. Bar, 20 µm. (D) Semiquantitative confocal microscopy analysis (n = 5 per group) of P1a levels in MDL-28170- relative to DMSO (solvent)-treated samples (100%, red broken line) using identical image settings. Note that the mean intensities of P1a fluorescence signals had increased ∼2-fold (compared with untreated skin) after treating *Plec^Ogna/+^* mice for 5 days with MDL-28170. Box and whisker plot as in Figure 2. P<0.01, unpaired Student's t test. (E) Immunoblotting of tissue extracts prepared from tail skin epidermis using antibodies to calpain-1. Note partial inhibition of autoproteolytic calpain-1 cleavage upon topical treatment of *Plec^Ogna/+^* mouse tails with MDL-28170.

To demonstrate the direct involvement of calpains in degradation of Ogna P1a, we incubated *Plec^Ogna/Ogna^* keratinocyte cultures with calpain inhibitors and monitored P1a levels by immunoblotting of cell lysates, at various timepoints of the drug treatment. Using the reversible calpain inhibitor ALLN, increased levels of P1a protein were observed as soon as 6 hours after application of the drug (Figure 11A), and a further ∼1.5-fold increase was detectable after 24 hours. At the same time, no autoproteolytically cleaved calpain-1 could be detected (Figure 11A), suggesting that only a small fraction of calpain-1 was active in these cells. Increased levels of P1a protein were also observed upon treatment of *Plec^Ogna/Ogna^* keratinocytes with the irreversible cysteine protease inhibitor E64d as well as the reversible calpain-specific inhibitor MDL-28170 (data not shown). Furthermore, we found that the exposure to MDL-28170 significantly increased the percentage of cells having formed HPCs (Figure 11B), correlating with the observed increase in P1a levels upon calpain inhibition.

To assess whether P1a expression could be restored in vivo, we topically applied MDL-28170 onto the tail skin of *Plec^Ogna/+^* mice over a period of five days and then measured P1a expression levels using confocal laser scanning microscopy of frozen tail sections. We found that compared to untreated control samples the P1a-specific immunofluorescence signal was clearly increased at the basal cell membrane of basal epidermal keratinocytes after this period (Figure 11C). The P1a levels in treated skin were estimated to correspond to ∼50%–60% of wild-type levels (Figure 11C). In a semiquantitative comparison (n = 5 per group, performed with a confocal microscope using identical image settings, we found a statistically significant (∼2-fold) increase in the P1a immunofluorescence signal intensity after MDL-28170 treatment (P<0.01; Figure 11D). Immunoblotting of epidermal extracts prepared from MDL-28170-treated mice revealed also a significant inhibition of autoproteolytic cleavage of calpain-1 relative to untreated controls (Figure 11E). In all, these data clearly demonstrated the involvement of calpains in P1a degradation in the skin of EBS-Ogna mice.

## Discussion

### A mouse model for EBS-Ogna, a disease of HDs

As an essential component of HDs, plectin connects ITGα6β4 to the keratin IF network, and failure to do so compromises HD performance [9], [17]–[19], [45], [46]. Exploiting the property of the EBS-Ogna mutation to specifically target hemidesmosomal plectin we have developed an EBS-Ogna knock-in mouse line to study the contribution of plectin to HD stabilization. The knock-in mouse faithfully replicates key features of the human disease, including the development of microlesions and skin fragility. Furthermore, as is distinctive for EBS-Ogna patients, the mutant mice did not develop muscular dystrophy, contrary to patients suffering from EBS-MD, the most common form of plectin-associated EBS. Our analysis showed that epidermal detachment in response to mechanical stress resulted from reduced numbers of HDs and compromised attachment of keratin IFs to their inner plate structures. Consistent with a dominant-negative effect of the EBS-Ogna mutation, morphometric and functional analysis of HDs did not reveal any statistically significant differences between hetero- and homozygous mice. The defects in HD formation could be traced to the degradation of P1a, the isoform that is recruited to HDs via binding to the integrin subunit β4. The EBS-Ogna mutation, affecting only P1a but not P1c and resulting in an exclusive HD phenotype, emphasizes the highly specific functions of the different plectin isoforms. Another dramatic example is a recently discovered mutation in plectin exon 1f that results in Limb-Girdle muscular dystrophy without skin involvement [13]. The fact that EBS-Ogna mice live to adulthood makes them not only a reliable animal disease model but also a model organism for studying HD homeostasis in adult stratified epithelia, with potential implications for several other types of EB as well as EB-independent diseases with HD-involvement, including bullous pemphigoid and squamous cell carcinoma [47], [48].

### Plectin 1a, but not BPAG1e, is required for efficient HD formation

HDs in stratified epithelia apparently have evolved to contain two members of the cytolinker protein family, plectin and BPAG1e. Interestingly, anchorage of keratin IFs at HDs was found to be compromised in mice carrying null alleles of either one of these proteins, indicating that their functions in HDs were similar but non-redundant [9], [49]. In contrast to humans and mice with BPAG1-deficiencies [49]–[51], in plectin-null mice as well as EBS-MD and EBS-PA patients, HD numbers were found to be reduced [9], [45], [52], demonstrating a specific role of plectin in HD formation. These observations are in line with the currently proposed mechanism of HD assembly, where in the initial step plectin-mediates the anchorage of laminin-clustered ITGβ4 to the keratin IF network, followed by the recruitment of BPAG2 and BPAG1e [24]. Our studies using EBS-Ogna mice refine this model by showing that P1a is the plectin isoform relevant for HD formation. The presence of HD-related structures lacking BPAG1e and BPAG2 in simple epithelial tissues [53], [54] suggests that the incorporation of both BPAG proteins into skin HDs occurred to meet the greater requirement of this tissue for mechanical stress protection.

### Lateral self-association of RDs: A specific feature of HD-associated plectin?

We have previously shown that in solution, native plectin can self-associate into network-like higher-order structures via interactions of plectin's globular end domains [30]. In addition, as we demonstrate here, the RDs of both wild-type and Ogna plectin are capable of oligomeric self-assembly via lateral alignment and association of their α-helical domains. Based on our findings we envisage plectin molecules to occur inside basal keratinocytes mainly in two oligomeric states. In the interior of the cell, plectin forms dimers, consistent with the shapes and dimensions of cytoskeleton-associated plectin molecules visualized by electron microscopy [55]. However, at the basal cell membrane, the recruitment of P1a to laminin-clustered ITGα6β4 molecules in developing HDs could lead to the proximal alignment of plectin's RDs, favoring their interaction and the assembly of compact highly ordered plectin structures with complexities going far beyond that of dimers (see working model, Figure 8G). Thus, ITGα6β4-induced oligomerization of dimeric plectin molecules by lateral association of their RDs could literally add another dimension to plectin's HD-stabilizing potential. This model offers an explanation why the Ogna mutation impairs plectin's HD-related functions, but not those that apparently do not require oligomerization, such as the organization of the keratinocyte IF network cytoarchitecture (Figure 5C). In this scenario, sufficient numbers of stable keratin-IF-linked HDs would only form if enough intact full-length P1a molecules are available for oligomerization. The greatly compromised formation of HDs in skin and cultured keratinocytes from EBS-MD patients, which lack expression of full length plectin molecules containing the RD [18], [45], [56], lend further support to this model. Whether other cytolinker proteins structurally related to plectin are capable of lateral association as well, and whether plectin can form hetero-oligomers with other cytolinker proteins remains to be shown. Hetero-oligomer formation has previously been observed in the case of periplakin and envoplakin [57], while desmoplakin has been reported to assemble exclusively into dimers [58].

### Targeted proteolysis of plectin 1a: A key to EBS-Ogna pathology

Even though our data showed that the Ogna mutation causes a localized structural defect in the coiled-coil domain of plectin, the major detectable and measurable difference between wild-type and Ogna RDs was that in the presence of keratinocyte proteases the mutant version was more susceptible to proteolysis. High sensitivity to proteolysis during cell lysis is characteristic for plectin [59], [60], and various proteases capable of plectin cleavage have been described, including caspases 2, 3, 6, 7, and 8 [32], [33], and calpain-1 [34]. Using an in vitro system to detect epidermis-specific protease activities which were able to cleave or even degrade plectin's RD, we found that the Ogna mutation sensitizes the plectin rod to degradation via the action of calpains and serine protease activities present in the epidermis. In accordance with an earlier report [34], we found that plectin, both as full-length version purified from cultured cells as well as in the form of the recombinant RD, can be cleaved by purified calpain-1. Moreover, we could detect increased P1a levels in cultured Ogna keratinocytes, as well as in skin of Ogna knock-in mice, after treatment with calpain-specific inhibitors, indicating that calpains are involved in the degradation of mutant P1a in vivo.

Calpains are a family of Ca^2+^-dependent cysteine proteases, which are expressed in a variety of tissues and are associated with numerous human pathologies [61]. Expression of calpain-1 has been detected in all cell layers of human epidermis [62], and its activity has been shown to increase in cultured human keratinocytes at confluence and during Ca^2+^-induced terminal differentiation [62], [63]. We found that in mouse epidermis, calpain-1 is predominantly expressed in the basal and granular keratinocyte cell layers. The low level of calpain-1 expressed in the spinous epidermal cell layers could be due to antigen masking, as has been reported for human skin [62]. We also found that calpain-1 partially colocalized with ITGβ4 at the basal cell membrane of basal keratinocytes, thus fulfilling the spatial requirements for a potential HD regulator. Indeed, we found that ionomycin-induced activation of calpains in cultured mouse keratinocytes caused the degradation of ITGβ4 and P1a, leading to the destruction of HDs. ITGβ4 has previously been shown to be intracellularly processed by calpains during Ca^2+^-induced terminal differentiation of keratinocytes [64]–[66]. Interestingly, however, in the presence of ionomycin, wild-type P1a displayed much slower degradation kinetics compared to ITGβ4, demonstrating that wild-type P1a incorporated into HPCs is fairly resistant to calpain-mediated proteolysis. Proteolytic degradation of hemidesmosomal components could be part of a cellular program to promote the detachment of keratinocytes from the basement membrane during the initial steps of differentiation [65]. In support of this hypothesis we observed that downregulation of ITGβ4 and P1a during Ca^2+^-induced terminal differentiation [25] correlated with an increase in the levels of autoproteolytically cleaved calpain-1 (unpublished data). It remains to be shown, however, whether a similar mechanism is operative in the epidermis in vivo.

Considering that only P1a is recruited to HDs, while P1c predominates in suprabasal cell layers and at lateral and inter-hemidesmosomal basal cell membranes (Figure 3), a close spatial relationship of calpain-1 with HDs would explain why P1c levels are unaffected in Ogna keratinocytes. It would also be in line with the observed downregulation of P1a and ITGβ4 (but not of P1c) along with the demolition of HDs during Ca^2+^-induced terminal differentiation of keratinocytes [25]. Although three calpain isoforms are expressed in skeletal muscle [67], we did not observe degradation of plectin's RD upon incubation with quadriceps muscle extracts. Studies in humans and rats have shown that activation of calpain-1 and calpain-3 is tightly regulated in skeletal muscle, and that even under exhaustive sprint- or endurance exercise both calpain isoforms remain predominantly in their autoproteolytically uncleaved, inactive form [68], [69]. In accordance with these findings, we detected autoproteolytically cleaved calpain-1 only in epidermis- but not skeletal muscle-derived protein extracts (unpublished data). Similar to skeletal muscle, calpain activation in other tissues unaffected by the Ogna mutation, including cerebral cortex and hippocampus, has been reported to be strongly suppressed [70]. This would explain why in EBS-Ogna mice and patients, plectin is only degraded in the epidermis.

The identity of the epidermis-specific plectin-degrading serine protease activity remains elusive at the moment, as multiple serine proteases are active in the epidermis (reviewed in reference [71]). It is also unclear what triggers the proteolytic attack on plectin's RD. A conformational change within the RD leading to the surface exposure of otherwise cryptic hydrophobic residues (including the mutation-inherent tryptophan) appears likely.

Based on our data we propose the following pathomechanism for EBS-Ogna. The mutated residue locally alters the conformation of plectin's dimeric RD by unfolding its coiled-coil structure around the site of the mutation. This renders plectin more susceptible to localized proteolytic degradation by basal cell membrane-associated keratinocyte proteases including calpains. When HD-recruited P1a is locally degraded by these proteases, its reduced level is insufficient to promote formation of HDs in adequate numbers and of optimal stability for keratin IF network anchorage, ultimately resulting in skin fragility in response to mechanical stress.

## Materials and Methods

### Ethics statement

This study was carried out in strict accordance with Austrian Federal Government laws and regulations. Protocols were approved by the Austrian Federal Ministry for Science and Research. All efforts were made to minimize suffering of animals.

### Generation of *Plec* ^Ogna/+^ mice

Generation of the targeting construct is described in Figure S1. The linearized targeting vector containing the EBS-Ogna mutation (c.5995C>T in RefSeq NM_011117.2, GenBank) was electroporated into embryonic stem cells (line E14.1 [72]). G418-resistant colonies of cells heterozygous for the correctly targeted allele [*Plec* ^Ogna/+^ (neo^r^)] were isolated and identified by Southern blot analysis of genomic DNA. The presence of the Ogna mutation was confirmed by sequence analysis. To generate chimeric mice, two recombinant ES cell clones (10–15 cells) were injected into blastocysts (isolated from C57BL/6 mice at day 3.5 postcoitum; p.c.) that were subsequently transferred into pseudopregnant C57BL/6×CBA females (3.5 day p.c.). Germ line chimeras obtained were bred to C57BL/6 females to obtain F1 mice heterozygous for the Ogna mutation [*Plec* ^Ogna/+^ (neo^r^)/+]. Heterozygous mice were bred to Cre deleter mice and the offspring screened for the excised neo^r^ cassette using Southern blot analysis of tail DNA by digestion with HindIII. Wild-type, heterozygous and homozygous littermate mice used in experiments were obtained by mating heterozygous animals.

### Tape stripping, TEWL, and dye penetration assay

Tape stripping, measurement of transepidermal water loss (TEWL), and dye penetration assays were performed as previously described [17].

### Topical application of MDL-28170

The cell-permeable calpain inhibitor MDL-28170 (Enzo Life Sciences) was dissolved in 100% (v/v) DMSO to obtain a 40 mg/ml stock solution. Prior to topical application, this stock solution was diluted 1∶10 in 100% (v/v) DMSO (final concentration of MDL-28170: 10.5 mM). Ten 4–5 months old female *Plec* ^Ogna/+^ mouse littermate pairs were divided into two groups of five mice. To the animals of one group, 100 µl of MDL-28170 solution was topically applied twice a day to a ∼2 cm-long section of their tail. The other group was similarly treated with the solvent [100% (v/v) DMSO] alone. Mice received a total of 10 treatments over a period of five consecutive days. They were killed by cervical dislocation 2 hours after the last treatment. The central ∼1 cm-long part of the tail segment treated was isolated, snap-frozen in isopentane (cooled with liquid N_2_), and processed for semiquantitative immunofluorescence microscopy analysis.

### Cell culture, transient transfection, and osmotic shock assay

Isolation of primary keratinocytes followed established protocols [73]. Keratinocytes from newborn or adult mice were cultured in keratinocyte growth medium (KGM) (Lonza) supplemented with 2, or 8% chelex-treated FCS (Sigma), respectively. The final concentration of Ca^2+^ in the growth medium was adjusted to either 0.05 (KGM/0.05) or 0.3 mM (KGM/0.3). All experiments were performed with keratinocytes isolated from littermate animals. Immortalized (p53-deficient) basal keratinocytes were derived from *Plec* ^+/+^/*p53* ^−/−^ and *Plec* ^−/−^/*p53* ^−/−^ mice [6], and were subcultured in KGM/0.05. These cells were used at passage numbers 10–15. For transient transfections we used FuGene (Roche). Osmotic shock stress assays were performed as previously described [27].

### Scratch wound closure assay

Scratch wound closure assays were performed as already described [26] using primary keratinocyte cell cultures. To prevent differentiation, cells were grown in KGM/0.05. 16 hours after induction of scratch wounds, cells were fixed with methanol and processed for immunofluorescence microscopy using anti-actin and anti-pan-keratin antibodies. Images were generated using a fluorescence laser-scanning microscope (LSM 510, Carl Zeiss MicroImaging) equipped with a Plan-Apochromat 10x/0.45NA objective lens. The average number of keratin-positive cells that had migrated into the scratch after 16 hours was counted in 3 randomly chosen fields along the scratch wound.

### Live cell imaging

Time-lapse video microscopy was performed using an AxioObserver Z1 microscope coupled to AxioCam MRm (Carl Zeiss MicroImaging) equipped with phase contrast optics. Cells were plated at a density of 2.5×10^5^ cells/cm^2^ and kept in KGM/0.3 during the whole period of observation. Migrating cells ectopically expressing GFP-tagged wild-type and Ogna mutant full-length P1a, or GFP alone, were monitored in parallel in a PM S1 incubator (Carl Zeiss MicroImaging) at 37°C and 5% CO_2_ using the “mark and find” module of AxioVision (version 4.8.1) image analysis software. Recordings of migrations started 6 hours after plating, and frames were taken with an EC Plan-Neofluar 10x/0.3NA objective lens in 10 min intervals over a period of 12 hours. Images were processed with Zeiss AxioVision 4.8.1 image analysis software and further analyzed with ImageJ (NIH) for manual tracking of migrating cells. To track the whole cell trajectory, cell nuclei were marked for each frame throughout the entire time lapse sequence. Cells that did not spread, entered mitosis, or migrated out of the field of view during the period of observation, were not taken into account for measuring. For statistical evaluation, we analyzed 30–40 cells per expressed cDNA construct in three independent experiments.

### Immunofluorescence and phase contrast microscopy of tissue sections and cultured cells

Tissues were fixed in 4% (w/v) paraformaldehyde in PBS and paraffin-embedded, or snap-frozen in isopentane (cooled with liquid N_2_), sectioned (2 µm) on a cryomicrotome, and fixed in acetone at −20°C [74]. Cultured cells were methanol–fixed (2 min, −20°C). After immunostaining and mounting in Mowiol, samples were examined using a LSM 510 microscope equipped with Plan-Apochromat 40x/1.3NA, Plan-Apochromat 63x/1.4NA and Plan-Apochromat 100x/1.4NA objective lenses. Digital images were processed using LSM 5 image browser and Adobe software package.

### Quantification of HPC formation in cultured keratinocytes

Cultured keratinocytes were fixed with methanol and processed for immunofluorescence microscopy using anti-ITGα6 and anti-pan-plectin antibodies. Images were generated using a LSM 510 microscope equipped with a Plan-Apochromat 63x/1.4NA objective lens. Cells displaying codistribution of ITGα6 and plectin in dense clusters at the basal cell surface were scored as HPC-positive. Cells that did not fulfill this criterion were scored as HPC-negative.

### Semiquantitative confocal immunofluorescence microscopy analysis

Confocal microscopy-based semiquantitative measurements of P1a immunofluorescence signal intensities at the basal cell membrane of basal epidermal keratinocytes was performed using cyrosections of mouse tail skin following the protocol given in [31]. A total of 3–5 cryosections per genotype or group was immunostained for P1a, and stacks of 6 sections (scan zoom, 1.0; z distance, 0.42 µm) of monochrome 12-bit gray-level images were recorded with a LSM510 confocal microscope equipped with a Plan-Apochromat 40x/1.3NA objective (Carl Zeiss MicroImaging), using identical settings and multichannel acquisition after excitation at 364 nm (DAPI; emission LP, 385 nm) and at 543 nm (RRX; emission BP, 560–615 nm). The pinhole was adjusted to 1 airy unit (corresponding to an optical slice thickness of 1.1 µm), and images with 2,048×2,048 pixels (0.11 µm isotropic) were recorded. Data analysis was performed with the full version of the Zeiss LSM software (version 4.2). The image plane with the highest mean intensity signal was chosen for analysis. Mean fluorescence signal intensities were obtained by measuring 4 independent dermo-epidermal junction zone areas (defined as n = 1) and subtracting the intensity of an adjacent area of the same size as background. Relative fluorescence intensities were calculated based on mean intensities measured for non-drug-treated or *Plec* ^+/+^ mice.

### Transmission electron microscopy and morphometric analysis of HDs

Five adult littermate mice per genotype were sacrificed by cervical dislocation, and skin samples excised from foot pads were fixed in 3% (w/v) glutaraldehyde in Sorensens buffer, pH 7.5, at 4°C overnight. Postfixation was performed in OsO_4_, followed by dehydration and embedding in epoxy resin [74]. Thin sections were cut with an ultramicrotome (Leica Microsystems), mounted on grids, contrasted with uranyl acetate and lead citrate, and examined at 80 kV in a transmission electron microscope (JEOL JEM-1210, Jeol Ltd.). Images were acquired using a Morada digital camera (Olympus SIS).

For morphometric analysis of HDs, 15 electron micrographs of the dermal-epidermal junction were obtained from each foot pad skin sample, corresponding to a total length of basal cell membrane analyzed in the range of 55–60 µm. A HD was defined as an area of electron dense material with a clearly discernible outer plate structure overlying the cytoplasmic side of the basal keratinocyte plasma membrane [45]. In each electron micrograph we measured the total length of basal cell membrane of basal keratinocytes as well as the length of each individual HD using iTEM and analySIS FIVE software packages (Olympus SIS). With these parameters we calculated for each electron micrograph the percentage of cross-sectioned basal cell membrane of basal keratinocytes containing HDs. Subsequently, the values obtained from all 15 electron micrographs were averaged, the averaged values from individual foot pad skin samples were pooled, and analyzed using two-way ANOVA. The average percentage of HDs with associated keratin-IF bundles was analyzed in a similar fashion by scoring similar numbers of HDs (∼600) per genotype for keratin-IF bundle attachment.

### Negative staining electron microscopy

Negative staining was done on carbon coated 400 mesh copper grids which were glow discharged prior to staining. Samples (8 µl in the respective buffer) were adsorbed to grids for one minute and then stained with 2% (w/v) uranyl acetate. After drying, the samples were viewed in a JEOL JEM-1210 transmission electron microscope at 80 kV.

### Molecular modeling

Three-dimensional models of native human plectin and of its p.Arg2000Trp mutant version (RefSeq NM_000445.3, GenBank) were built with the program MODELLER (version 9.9 of April 2011, http://salilab.org/modeller/) by means of sequence alignments generated manually and by using as templates the parallel dimeric coiled-coil of the PDB file 3NMD (chains A and B), or the antiparallel dimeric coiled-coil of the PDB file 2CCF (chains A and B). The program DeepView (release 4.02 of April 2011, http://spdbv.vital-it.ch/) was used for energy minimization. Coiled-coil tendency predictions were performed with COILS (www.ch.embnet.org/software/COILS_form.html), Multicoil (multicoil.lcs.mit.edu/cgi-bin/multicoil), and Paircoil2 (paircoil2.csail.mit.edu/paircoil2.html).

### Antibodies

A complete list of primary antibodies is given in Table S1 and Table S2. All secondary antibodies were purchased from Jackson Immuno-Research Laboratories.

### Immunoblotting and preparation of cell lysates and tissue extracts

Epidermis and dermis from tail skins of adult mice were separated by treatment with dispase (Roche, 18 hours, 4°C), snap-frozen in liquid nitrogen and pulverized. Epidermal tissues suspended in 1% (v/v) Triton X-100, 0.5% (v/v) NP-40, 150 mM NaCl, 20 mM Tris-HCl, pH 7.5, supplemented with complete protease inhibitor cocktail (Roche), were mechanically disrupted using a Polytron PT-3000 homogenizer (Kinematica). One volume of tissue homogenate was then combined with one volume of 5× SDS sample buffer, and after boiling for 5 min, insoluble material was removed by centrifugation at 13,000× g (15 min, RT). Cell lysates were prepared from cultures of post-confluent keratinocytes as described [26]. For calpain immunoblots, tissues were homogenized directly in 5× SDS sample buffer.

Aliquots of epidermal cell lysates or epidermal tissue homogenates were subjected to SDS-PAGE and, after immunoblotting using peroxidase-coupled secondary antibodies, protein bands were visualized by exposure to X-ray film. Quantitation of chemiluminescent protein bands was performed as described [75].

### Expression, purification, and characterization of recombinant proteins

DNA fragments encoding the rod of plectin with or without flanking sequences were generated by high fidelity PCR. The mutant (EBS-Ogna) rod was created by site-directed mutagenesis (QuickChange, Stratagene). Wild-type and mutant cDNAs were subcloned into the baculovirus expression vector pFBHT (Invitrogen) and a modified version of this vector carrying the GST tag instead of the His tag. Recombinant baculoviruses were generated using the Bac-to-Bac system (Invitrogen), transfected into Sf9 insect cells using CellFECTIN (Invitrogen), and high-titer baculovirus stocks were prepared. The recombinant viruses encoded wild-type and mutant versions of the RD (exon 31) of plectin (pNV10 or pNV14, and pNV11 or pNV15, respectively; Figure S6), and the rod flanked by spectrin repeat 9 and the beginning of the C-terminal domain (pHLH20/wt and pHLH20/Ogna, respectively; Figure 6A). For protein expression, Sf9 cells were grown in Insect Xpress medium (Lonza) supplemented with 10% FCS and 2 mM glutamine, infected with recombinant baculoviruses, and harvested 72 h post infection. Cells were lysed in 20 mM Hepes-HCl, pH 7.4, 1% (v/v) Triton X-100, 1% (v/v) glycerol, 1 mM PMSF (Hepes buffer). Following centrifugation at 16,000× g (15 min, 4°C), supernatants were purified by affinity chromatography using 1 ml HiTrap chelating HP or GSTrap HP columns with the Äkta FPLC system (GE Healthcare). His-tagged fusion proteins were bound to Co^2+^-charged columns and eluted with a linear gradient of 0–500 mM imidazole in Hepes buffer. GST-tagged proteins were bound in 1×PBS, and eluted with 50 mM Tris-HCl, pH 8.0, 10 mM glutathione. Fractions containing purified proteins were pooled, and their protein content quantified (Bradford reagent, BioRad), before being stored at 4°C. Blue native gel electrophoresis (BN-PAGE) was carried out in a discontinuous native gel electrophoresis system as described [76]. Samples were resolved in 4–10% polyacrylamide gradient gels.

### Size exclusion chromatography with multiangle laser light scattering (SEC-MALS)

The liquid chromatography equipment consisted of a HPLC system (Agilent Technologies) connected in series with a triple-angle laser light scattering detector (miniDAWN TREOS, Wyatt Technology), a UV detector at 280 nm (Agilent technologies) and a refractive index detector (RI-101, Shodex). Protein samples were eluted on a Superose 6 10/300 GL column (GE Healthcare) at a flow rate of 0.5 ml/min. The protein concentration was 2 mg/ml; the sample load 75 µl. For data analysis Astra software (Wyatt technology) was used.

### Chemical crosslinking and assessment of oligomer dissociation

Purified proteins (10 µg) in 20 mM Hepes-HCl, pH 7.4, were mixed with 2 µl freshly prepared DMS (20 mg/ml, same solution) in a total volume of 20 µl. The reaction was allowed to proceed for 1 h at RT and stopped by addition of 6× SDS sample buffer followed by boiling for 3 min. Samples were analyzed by SDS-5% PAGE. Plectin-enriched IF fractions prepared from glioma C6 cells [30] were run in parallel using plectin as high molecular mass marker (∼500 kDa).

Dissociation of plectin rod oligomers to monomers in solution was monitored by SDS-PAGE. Aliquots of plectin in 20 mM Hepes-HCl, pH 7.4, were incubated at different temperatures or with increasing concentrations of urea, cross-linked with DMS, and samples analyzed by SDS-5% PAGE. Experiments performed in duplicates were repeated three times. Gels were stained with Coomassie Brilliant Blue R-250, scanned, and the relative quantities of monomeric and oligomeric forms estimated by densitometry analysis using Quantiscan software (Biosoft).

### In vitro degradation assays

Epidermal protein extracts for in vitro degradation assays were prepared in 20 mM Tris-HCl, pH 7.5, 300 mM NaCl, 0.2 mM DTT as described [77]. The resulting epidermal homogenate was centrifuged at 10,000× g for 30 min (4°C), and the supernatant collected. 30 µl of this extract (∼5 µg of protein) were incubated with 10 µg GST-tagged wild-type or Ogna mutant rod protein in a total volume of 40 µl at 37°C for the times indicated in the text. Where indicated, incubations alternatively contained 2 µl DMSO, 50 µM MDL 28170 (Sigma-Aldrich), 5 µM ALLN (N-Acetyl-Leu-Leu-Nle-CHO, Calbiochem), 20 µM MG132 (Z-Leu-Leu-Leu-al, Sigma-Aldrich), 10 or 100 nM Bortezomib (LC Laboratories), 20 mM EDTA, or 2 mM PMSF (Sigma-Aldrich). 10 µl of 5× SDS sample buffer were added to stop the reactions, followed by incubations at 95°C for 5 min. Samples were analyzed by SDS-8% or -10% PAGE. Experiments were repeated at least three times, using two independently prepared epidermal protein extracts. To determine degradation kinetics of wild-type versus mutant rod proteins, immunoblots from three independent in vitro degradation experiments were scanned, and the relative quantities of the intact rod proteins estimated by densitometry analysis using Quantiscan software (Biosoft).

### Statistics

Comparisons between values of two groups were made using unpaired Student's t-test (alpha = 0.05). Normal distribution of values was analyzed using the D'Agostino & Pearson omnibus K2 normality test (alpha = 0.05) or the Anderson-Darling normality test (alpha = 0.05). Equality of variances was analyzed using Bartlett's test (alpha = 0.05). Comparisons among values of multiple groups were performed using analysis of variance (ANOVA) to determine the overall significance (alpha = 0.05). The significance between the individual groups was subsequently determined using the Tukey-Kramer post test for multiple comparisons (alpha = 0.05). For two-factorial comparisons among values of multiple groups we used two-way ANOVA to determine the overall significance (alpha = 0.05). In cases were mice were assessed repeatedly, we used a repeated-measurements two-way-ANOVA. The significance between the individual groups in two-way ANOVA was subsequently determined using the Bonferroni post test for multiple comparisons (alpha = 0.05). All statistical analyses were performed using GraphPad Prism 5.

## Supporting Information

Figure S1 Targeting strategy and molecular analysis of Ogna knock-in mice. (A) Schematic maps of the targeting construct, wild-type plectin locus (*Plec* ^+^), and targeted alleles (*Plec* ^Ogna(neor^); *Plec* ^Ogna^) with relevant restriction sites (X, XmaI; H, HindIII). Relevant exons (numbered black boxes), loxP sites (empty triangles), the Ogna mutation (star), mutant allele probes, and sizes of HindIII restriction digests are indicated. The targeting construct, a 15.5 kb-long fragment of the plectin gene starting at exon 15 and finishing in exon 32, carried the Ogna mutation in exon 31 and a loxP-flanked neomycin resistance (neo^r^) cassette (white box) located in the intron between exons 31 and 32. (B) Southern blot analysis of HindIII-digested genomic DNA from mutant (*Plec^Ogna(neor)/+^*; *Plec^Ogna/+^*) and wild-type mice (*Plec^+/+^*). The probe recognizing the neo^r^ cassette detected a 19.6-kb band only before removal of the neo^r^ cassette. The 5′ and 3′ external probes detected a 30-kb band from the wild-type allele, and a 12- or 18-kb band from the Ogna allele. (C) PCR genotyping of mouse genomic DNA. DNA amplification using primers flanking the remaining loxP site yielded 204-bp and 270-bp DNA fragments from the wild-type and Ogna alleles, respectively. (D) Sequencing analysis of genomic PCR products spanning the Ogna mutation. Chromatogram shows the DNA area flanking the mutation. (E) RT-PCR analysis of total RNA isolated from several tissues and primary keratinocytes from *Plec* ^Ogna/+^ mice. The primers were designed to amplify a cDNA fragment of 1800 bp which is visible in all the tissues tested as well as in primary keratinocytes. To amplify exclusively cDNA, the reverse primer spanned an exon/intron border. Size marker, 1-kb DNA ladder. (F) cDNA sequence of plectin mRNA transcripts expressed in *Plec* ^Ogna/+^ skin. Sequencing of the RT-PCR product shown in (E) confirmed the presence of two transcripts corresponding to the wild-type and Ogna mutant alleles. Arrows in D and F point to the C/T heterozygosity.
(TIF)

Figure S2 Immunolocalization of K5 on frozen sections of leg skin from wild-type (+/+) and mutant mice. (A–C) 1-day-old mouse pubs; (D–F) 3-month-old mice. Note unaltered K5 expression in mutant compared to *Plec* ^+/+^ epidermis. Arrowheads, basal cell membrane of basal keratinocytes. Bar, 50 µm.
(TIF)

Figure S3 Normal plectin expression in skeletal muscle tissue of Ogna mice. (A) Immunoblotting (SDS-6% PAGE) of quadriceps muscle cell lysates using antiserum to plectin. Non-contiguous lanes run on the same gel were spliced together as indicated. (B) Coomassie blue-stained SDS-10% polyacrylamide gel used for normalizing gel loading.
(TIF)

Figure S4 Compromised ex vivo formation of HPCs in Ogna keratinocyte cell clusters and stratified monolayers. Primary keratinocytes isolated from newborn mice were grown in KGM/0.3 until post-confluence. (A,B) Immunolocalization (double labeling) of ITGα6 and plectin in cell clusters formed after 2 days in culture (A) and in stratified cell monolayers at 2 days post-confluence (B). Composite images were generated from confocal stacks by maximum intensity projections of the three optical sections closest to the substrate level. Note, in clusters of wild-type (+/+) keratinocytes (A, upper row), ITGα6 and plectin show codistribution in densely clustered HPCs (arrowheads) contrasting the more diffuse distribution in Ogna keratinocytes (A, lower row); in stratified cell monolayers, HPCs (B, arrowheads) are reduced in basal keratinocytes of wild-type, and hardly detectable in Ogna cells. Bars, 50 µm.
(TIF)

Figure S5 Wild-type and Ogna P1a both can rescue the aberrant keratin network mesh size of plectin-null keratinocytes. (A–F) Plectin-null (*Plec* ^−/−^) keratinocytes transfected with expression plasmids encoding GFP-tagged full-length P1a with (Ogna P1a) or without (wt P1a) the Ogna mutation, or just GFP (mock), were fixed and immunolabeled for K5 and GFP. (D–F) K5 immunofluorescence images were contrast-enhanced by conversion to grey scale and inversion of contrast. Note the more delicate (filamentous) K5 IF network appearance upon forced expression of either wt or Ogna P1a compared to untransfected cells (arrows in E and F) or cells expressing GFP alone (D). (G) Rescue efficiency was determined by analysis of >100 plectin-null keratinocytes transiently expressing either wt P1a, Ogna P1a, or GFP (mock). Keratinocytes with average filament-filament distances of below 1.5 nm were considered rescued. The column diagram shows the average percentage of rescued cells of three independent experiments. Data are presented as mean, error bars represent 95% CI. ** P<0.01 (one-way ANOVA with Tukey post test for multiple comparisons). Bar, 20 µm.
(TIF)

Figure S6 Keratin IF networks of *Plec* ^Ogna/+^ keratinocytes are more sensitive to hypo-osmotic shock compared to *Plec* ^+/+^ cells. Column diagram shows proportions (%) of cells with collapsed K5 IF networks in *Plec* ^+/+^ and *Plec* ^Ogna/+^ keratinocytes with or without urea treatment. Data shown represent mean values (±95% CI) from cell counts (>120/genotype) in randomly chosen optical fields of six independent experiments. Urea-induced hypo-osmotic shock caused massive keratin collapse in *Plec* ^Ogna/+^ but not in *Plec* ^+/+^ keratinocytes. A trend towards increased K5 IF collapse in untreated *Plec* ^Ogna/+^ keratinocytes, statistically was not significant. *** P<0.001 (two-way ANOVA with Bonferroni post test).
(TIF)

Figure S7 The plectin Ogna rod can hetero-oligomerize with the wild-type rod and can form paracrystalline polymers. (A,B) Protein extracts from Sf9 cells, co-infected with baculoviruses expressing His-tagged wild-type and GST-tagged Ogna-rod versions, were incubated with glutathione Sepharose. The Sepharose-bound GST-Ogna rod and its associated proteins were recovered by centrifugation, washed three times, and eluted with 5x-SDS sample buffer. Eluates were resolved by SDS-8%-PAGE (A) and analyzed by immunoblotting using anti-His-tag antibodies (B). Lanes 1, input; 2, wash fractions; 3, pull-down eluates. Positions of GST- and HIS-tagged rod proteins are indicated. Note, both rods can also form homo-oligomers (Figure 6A) and thus only a fraction of HIS-wild-type rod is recovered in hetero-oligomeric form. (C–E) Electron microscopy of uranyl acetate-stained polymeric (paracrystalline) structures formed from plectin RDs containing the Ogna mutation. pHLH20/Ogna-encoded recombinant versions of plectin's RD were incubated in 50 mM sodium phosphate, pH 7.4, 300 mM NaCl, 170 mM imidazole, and 1 mM PMSF for 1 hour at 37°C before being processed for electron microscopy. Constrictions (D, arrows) of sheet-like structures may represent transition states between flat (collapsed) (C,D) and tube-like (E) structures. Note relative short length of polymers compared to wild-type (Figure 8). Bars, 100 nm. (F–H) Identical concentrations (0.15 mg/ml) of pHLH20/wt- and pHLH20/Ogna-encoded recombinant versions of plectin's RD (Figure 6A), processed in parallel, were adsorbed to 400 mesh grids, and grids subjected to uranyl acetate staining and electron microscopy. Images of 20 RD polymers were generated per protein from randomly selected fields. Three experiments were performed in total. (F) The average polymer length was not significantly different between wild-type (WT) and Ogna RDs, although a trend towards smaller polymer length was noticeable in case of the Ogna RD. Data are shown as mean values ±95% CI (n = 3). P = 0.067. (G) Scatter dot plot of all measured values pooled from three experiments indicating reduced numbers of Ogna RD polymers longer than 500 nm. Red horizontal bars show the mean, the thin broken line indicates the cutoff used for the statistical analysis shown in (F). (H) Statistical test of measured values shown in (G). Note reduced percentage of Ogna RD polymers larger than 500 nm. Data are shown as mean values ±95% CI (n = 3). Statistical significance between wild-type and Ogna RD polymer populations was demonstrated by unpaired Student's t-test (* P<0.05).
(TIF)

Figure S8 Quantitative analysis of plectin isoform transcripts in epidermal tissues. Total RNA was isolated from epidermis of *Plec* ^+/+^ and mutant mice using Trizol (Invitrogen). RNA (1 µg) was reverse-transcribed using SuperScript II (Invitrogen). Primer pairs were designed using Primer3 (http://frodo.wi.mit.edu/primer3). The amplicon ranged from 100 to 120 base pairs and spanned an intron. Reactions were performed in 20 µl final volume using SYBR Green I Master Mix (Roche) and processed on a Roche LightCycler 480. Data were analyzed using the software supplied with the instrument. Results originate from three independent experiments with each data point assayed in duplicate. RNA was isolated from two independent samples. (A) Relative quantification. The graph shows the relative expression levels of plectin exons in mutant versus wild-type epidermis. Quantification was done by the method of Pfaffl [78] using the housekeeping gene hypoxanthine guanine phosphoribosyl transferase 1 (HPRT1) for normalization. A ratio of 1 indicates no difference in expression between mutant and wild-type epidermis. (B) Absolute quantification. Results provide insights into the expression of selected plectin isoforms in the epidermis. Transcript copy numbers were calculated using a standard curve based on serial dilutions of the same exons cloned into plasmids. Note that P1c accounts for ∼80% of the total plectin expressed in the epidermis, while P1a represents a lesser fraction. This data are consistent with P1c expression in the basal as well as the suprabasal cell layers while expression of P1a is restricted to basal keratinocytes (see Figure 3, D–I). Columns represent the mean, error bars the SD.
(TIF)

Figure S9 In vitro digestion of plectin by calpain-1 and detection of serine protease activities in epidermal protein extracts. (A) Baculovirus-expressed GST-tagged wild-type rod protein (10 µg) in 30 µl of 20 mM Tris–HCl, pH 7.5, 25 mM NaCl, 1 mM β-mercaptoethanol (solution A) was pre-incubated with 1 mM CaCl_2_ at 25°C for 5 min before proteolysis was started by adding 10 µl of solution A containing 200 ng of purified human calpain-1 (Sigma-Aldrich), followed by incubation at 25°C for the indicated times. Samples were separated by SDS-10% PAGE, and intact plectin rod protein and cleavage products were detected by Coomassie blue staining. Note the appearance of multiple cleavage products (indicated by arrows and brackets) upon incubation with calpain-1. Similar cleavage patterns were obtained using His-tagged rod protein (data not shown). (B) Native full-length plectin (∼1 µg) purified from glioma C6 cells [79] was subjected to proteolysis as described in (A), except that only 50 ng instead of 200 ng calpain-1 were used. Samples were separated by SDS-5% PAGE, and intact plectin protein and cleavage products (arrows) were detected by immunoblotting using anti-plectin mAb 10F6. (C) Presence of gelatinolytic serine protease activities in epidermal tissue cell extracts analyzed by zymography. Protein extracts were prepared from epidermis as described in Materials and Methods, incubated with either 2 mM PMSF or 5 mM AEBSF (Sigma-Aldrich) to inhibit serine proteases, and then applied to zymography [80] in 12% polyacrylamide gels impregnated with 1 mg/ml gelatin. 15 µg of protein were loaded per lane. Note the presence of strong gelatinolytic activities at ∼52 and ∼24 kDa (arrows), which can be inhibited to a large extent by PMSF and AEBSF, demonstrating that they represent serine proteases. The ∼24 kDa serine protease activity could correspond to trypsin, the expression of which has been demonstrated in basal keratinocytes of skin in situ [81].
(TIF)

Figure S10 Expression of calpain-1 in mouse skin. (A) Immunolocalization of calpain-1 and ITGβ4 on frozen tail skin sections from adult wild-type mice. Strongest calpain-1 expression is detected in the basal (arrows) and granular (arrowheads) cell layers of the epidermis. Note prominent localization of calpain-1 at the basal cell membrane of basal keratinocytes. Two serial sections of four different wild-type specimens were examined, with no noticeable differences in staining pattern between samples. Bar, 50 µm. (B) Calpain-1 immunoblotting of cell lysates prepared from primary keratinocytes (pmk) (cultured in KGM/0.3) and of tissue extracts prepared from wild-type epidermis (epid.). Note, the 80 kDa (inactive) form of the protease is present in both, keratinocytes and epidermis, whereas the autoproteolytically cleaved (activated) 76 kDa form predominates in the epidermis. Asterisk, protein band of unknown identity.
(TIF)

Figure S11 Activation of calpains in cultured keratinocytes leads to degradation of hemidesmosomal proteins. (A–D) Subconfluent cultures of immortalized *Plec^+/+^* keratinocytes were maintained in KGM/0.05, before being switched to KGM supplemented with 1.8 mM CaCl_2_. Cells were then either left untreated or challenged (for the indicated times) with the Ca^2+^ ionophore ionomycin (5 µM) with or without the addition of the calpain inhibitor MDL-28170 (50 µM). (A) Immunoblotting of cell lysates using antibodies to proteins indicated. Note strongly decreased levels of calpain-1 due to ionomycin-induced autoproteolytic cleavage (indicative of calpain-1 activation), which was blocked by MDL-28170. Also note gradually decreasing P1a and ITGβ4 levels upon ionomycin challenge, which could be reversed by MDL-28170 treatment. The kinetics of P1a degradation were slower compared to that of ITGβ4, suggesting that P1a was more resistant to calpain-mediated degradation than ITGβ4. The (only) partial inhibition of P1a degradation with MDL-28170 observed after 5 hours of ionomycin treatment likely resulted from the activation of caspases at this timepoint (data not shown). (B,C) Densitometric quantifications of P1a (B) and ITGβ4 (C) protein levels relative to that in control samples (100%) using K5 as loading control. Mean values ±SEM (n = 4) are shown. Statistical significance was demonstrated by one-way ANOVA with Tukey posttest for multiple comparisons (* P<0.05, ** P<0.01, *** P<0.001). (D) Immunolocalization (double labeling) of ITGα6 (green) and plectin (red) in immortalized *Plec^+/+^* keratinocytes with or without ionomycin treatment for the times indicated. Nuclei were stained with DAPI (blue). Composite images were generated from confocal stacks by maximum intensity projections of the three optical sections closest to the substrate level. In untreated keratinocytes, ITGα6 and plectin show codistribution in densely clustered HPCs (arrowheads). After 1 hour of ionomycin challenge, HPCs start to become less densely clustered (arrowheads) and ultimately disappear after 5 hours. Bar, 20 µm.
(TIF)

Table S1 Primary antibodies used for immunofluorescence microscopy.
(DOC)

Table S2 Primary antibodies used for immunoblotting.
(DOC)

Video S1 Representative time lapse video showing migration of keratinocytes transiently expressing wild-type P1a. Migrating immortalized *Plec* ^+/+^ keratinocytes expressing wild-type P1a-GFP were monitored using time-lapse video microscopy. Images were collected every 10 minutes for 12 hours. Note that GFP-fluorescence was only visualized during the first time frame and is shown at the start of the video. Only cells strongly expressing full length P1a (arrows) were used for determination of single cell migration velocities. Cells that had not yet fully spread (arrowhead 1), or were expressing P1a only weakly (arrowhead 4) were not taken into account for measuring.
(AVI)

Video S2 Representative time lapse video showing migration of keratinocytes transiently expressing Ogna P1a. Migrating immortalized *Plec* ^+/+^ keratinocytes expressing Ogna P1a-GFP were monitored using time-lapse video microscopy. Images were collected every 10 minutes for 12 hours. Note that GFP-fluorescence was only visualized during the first time frame and is shown at the start of the video. Only cells strongly expressing full length P1a (arrows) were used for determination of single cell migration velocities. Cells that had not yet fully spread (arrowhead 1), migrated out of the field of view during the period of observation (arrowhead 2), were displaying signs of senescence (arrowhead 3), or were expressing P1a only weakly (arrowhead 4) were not taken into account for measuring.
(AVI)
