# Supplementary figures and images for: Targeted Proteolysis of Plectin Isoform 1a Accounts for Hemidesmosome Dysfunction in Mice Mimicking the Dominant Skin Blistering Disease EBS-Ogna
Source: PLoS Genet. 2011 Dec 1;7(12):e1002396. doi: 10.1371/journal.pgen.1002396 (PMC3228830; doi:10.1371/journal.pgen.1002396)

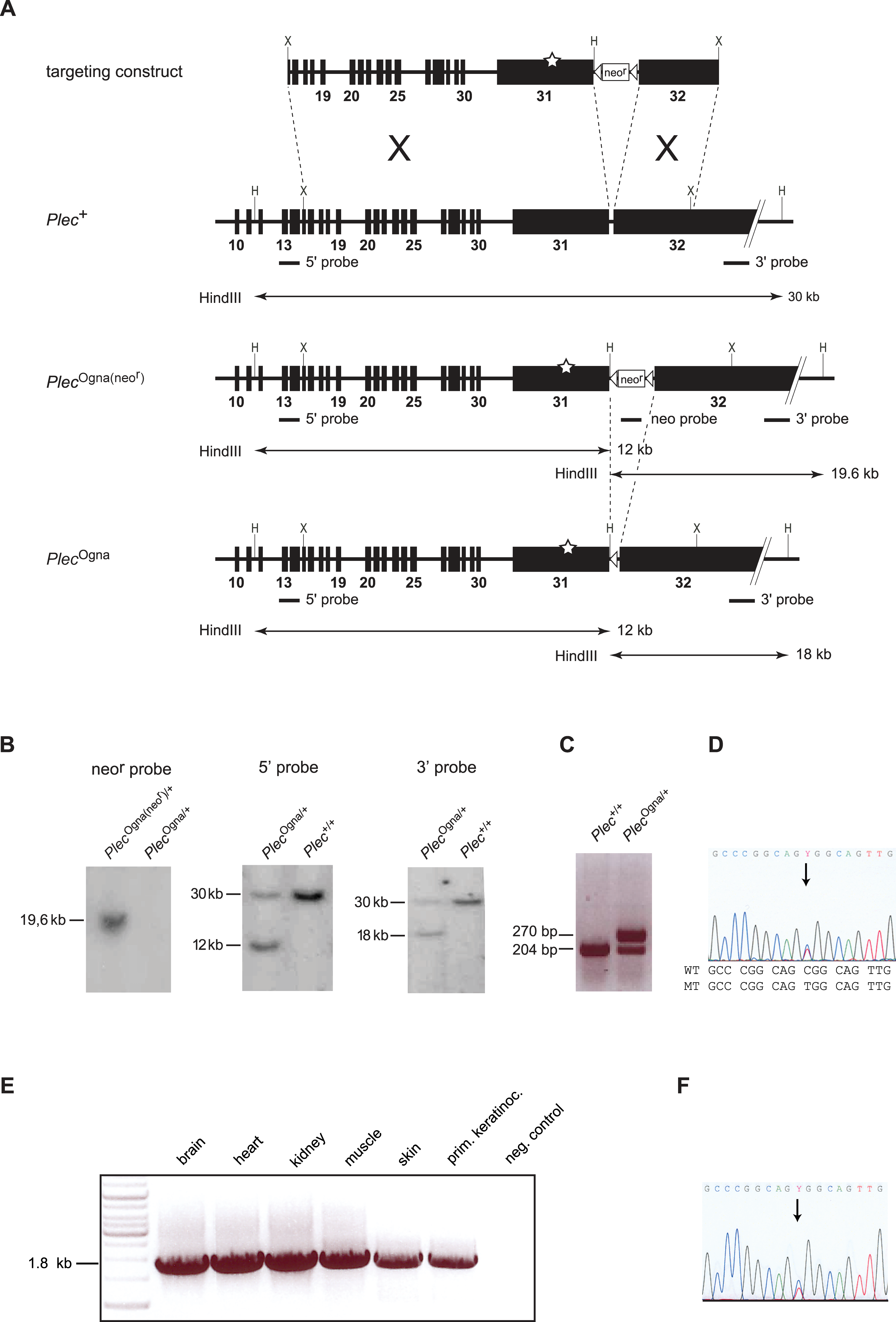

Supplement: Figure S1 — Targeting strategy and molecular analysis of Ogna knock-in mice. (A) Schematic maps of the targeting construct, wild-type plectin locus (Plec +), and targeted alleles (Plec Ogna(neor); Plec Ogna) with relevant restriction sites (X, XmaI; H, HindIII). Relevant exons (numbered black boxes), loxP sites (empty triangles), the Ogna mutation (star), mutant allele probes, and sizes of HindIII restriction digests are indicated. The targeting construct, a 15.5 kb-long fragment of the plectin gene starting at exon 15 and finishing in exon 32, carried the Ogna mutation in exon 31 and a loxP-flanked neomycin resistance (neor) cassette (white box) located in the intron between exons 31 and 32. (B) Southern blot analysis of HindIII-digested genomic DNA from mutant (PlecOgna(neor)/+; PlecOgna/+) and wild-type mice (Plec+/+). The probe recognizing the neor cassette detected a 19.6-kb band only before removal of the neor cassette. The 5′ and 3′ external probes detected a 30-kb band from the wild-type allele, and a 12- or 18-kb band from the Ogna allele. (C) PCR genotyping of mouse genomic DNA. DNA amplification using primers flanking the remaining loxP site yielded 204-bp and 270-bp DNA fragments from the wild-type and Ogna alleles, respectively. (D) Sequencing analysis of genomic PCR products spanning the Ogna mutation. Chromatogram shows the DNA area flanking the mutation. (E) RT-PCR analysis of total RNA isolated from several tissues and primary keratinocytes from Plec Ogna/+ mice. The primers were designed to amplify a cDNA fragment of 1800 bp which is visible in all the tissues tested as well as in primary keratinocytes. To amplify exclusively cDNA, the reverse primer spanned an exon/intron border. Size marker, 1-kb DNA ladder. (F) cDNA sequence of plectin mRNA transcripts expressed in Plec Ogna/+ skin. Sequencing of the RT-PCR product shown in (E) confirmed the presence of two transcripts corresponding to the wild-type and Ogna mutant alleles. Arrows in D and F point to the C/ [file pgen.1002396.s001.tif]

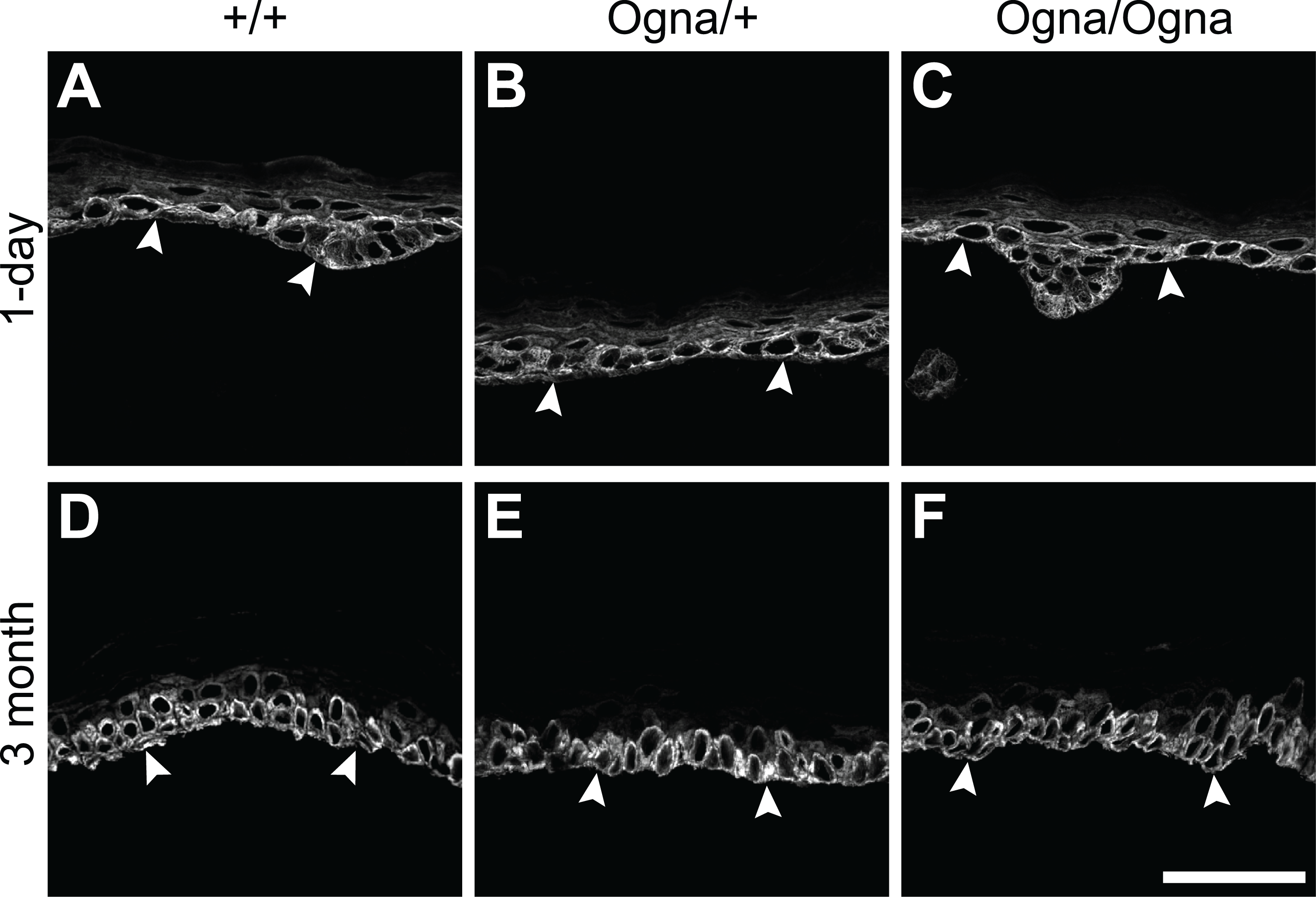

Supplement: Figure S2 — Immunolocalization of K5 on frozen sections of leg skin from wild-type (+/+) and mutant mice. (A–C) 1-day-old mouse pubs; (D–F) 3-month-old mice. Note unaltered K5 expression in mutant compared to Plec +/+ epidermis. Arrowheads, basal cell membrane of basal keratinocytes. Bar, 50 µm. (TIF) [file pgen.1002396.s002.tif]

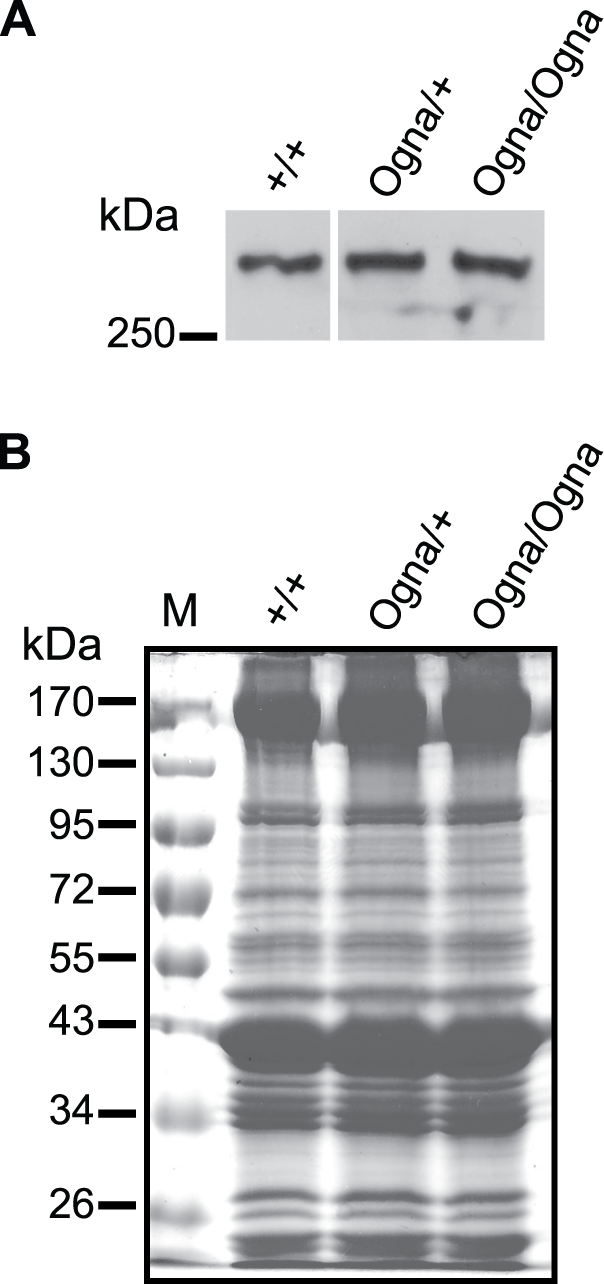

Supplement: Figure S3 — Normal plectin expression in skeletal muscle tissue of Ogna mice. (A) Immunoblotting (SDS-6% PAGE) of quadriceps muscle cell lysates using antiserum to plectin. Non-contiguous lanes run on the same gel were spliced together as indicated. (B) Coomassie blue-stained SDS-10% polyacrylamide gel used for normalizing gel loading. (TIF) [file pgen.1002396.s003.tif]

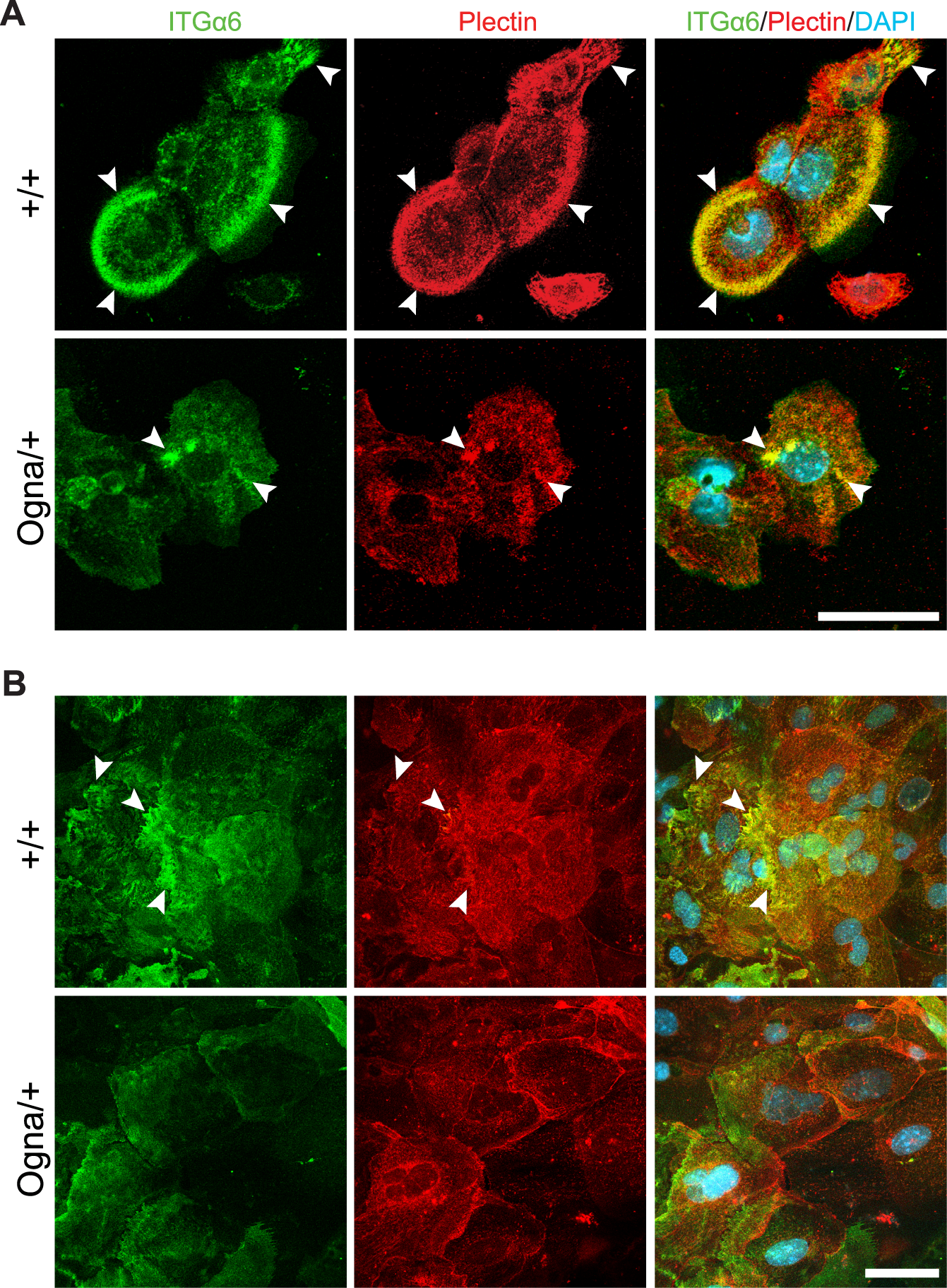

Supplement: Figure S4 — Compromised ex vivo formation of HPCs in Ogna keratinocyte cell clusters and stratified monolayers. Primary keratinocytes isolated from newborn mice were grown in KGM/0.3 until post-confluence. (A,B) Immunolocalization (double labeling) of ITGα6 and plectin in cell clusters formed after 2 days in culture (A) and in stratified cell monolayers at 2 days post-confluence (B). Composite images were generated from confocal stacks by maximum intensity projections of the three optical sections closest to the substrate level. Note, in clusters of wild-type (+/+) keratinocytes (A, upper row), ITGα6 and plectin show codistribution in densely clustered HPCs (arrowheads) contrasting the more diffuse distribution in Ogna keratinocytes (A, lower row); in stratified cell monolayers, HPCs (B, arrowheads) are reduced in basal keratinocytes of wild-type, and hardly detectable in Ogna cells. Bars, 50 µm. (TIF) [file pgen.1002396.s004.tif]

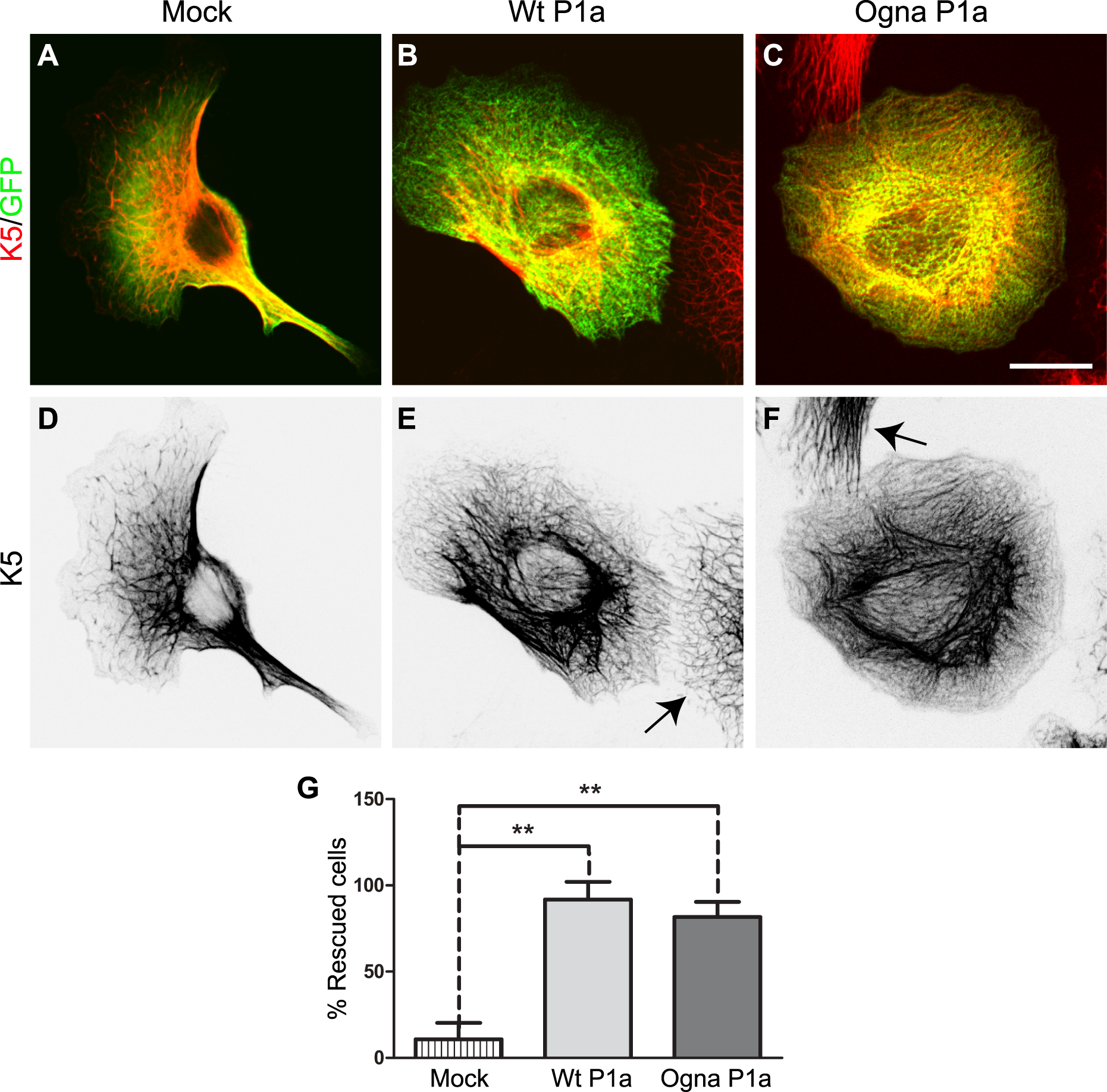

Supplement: Figure S5 — Wild-type and Ogna P1a both can rescue the aberrant keratin network mesh size of plectin-null keratinocytes. (A–F) Plectin-null (Plec −/−) keratinocytes transfected with expression plasmids encoding GFP-tagged full-length P1a with (Ogna P1a) or without (wt P1a) the Ogna mutation, or just GFP (mock), were fixed and immunolabeled for K5 and GFP. (D–F) K5 immunofluorescence images were contrast-enhanced by conversion to grey scale and inversion of contrast. Note the more delicate (filamentous) K5 IF network appearance upon forced expression of either wt or Ogna P1a compared to untransfected cells (arrows in E and F) or cells expressing GFP alone (D). (G) Rescue efficiency was determined by analysis of >100 plectin-null keratinocytes transiently expressing either wt P1a, Ogna P1a, or GFP (mock). Keratinocytes with average filament-filament distances of below 1.5 nm were considered rescued. The column diagram shows the average percentage of rescued cells of three independent experiments. Data are presented as mean, error bars represent 95% CI. ** P<0.01 (one-way ANOVA with Tukey post test for multiple comparisons). Bar, 20 µm. (TIF) [file pgen.1002396.s005.tif]

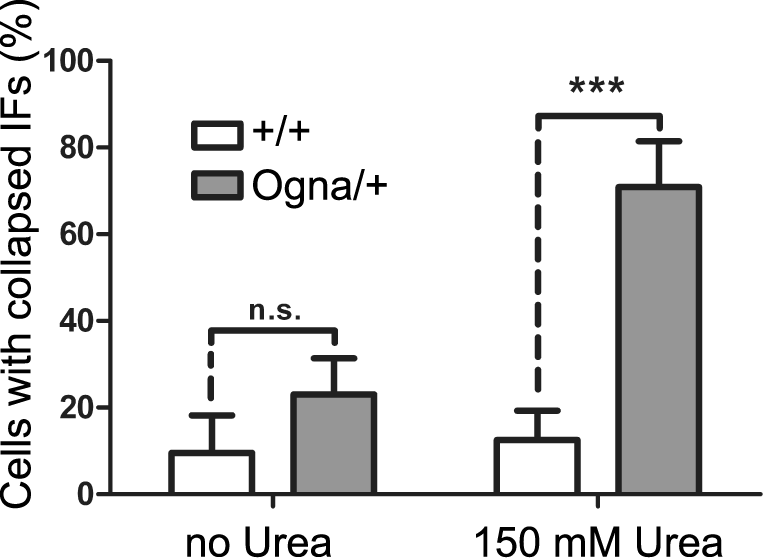

Supplement: Figure S6 — Keratin IF networks of Plec Ogna/+ keratinocytes are more sensitive to hypo-osmotic shock compared to Plec +/+ cells. Column diagram shows proportions (%) of cells with collapsed K5 IF networks in Plec +/+ and Plec Ogna/+ keratinocytes with or without urea treatment. Data shown represent mean values (±95% CI) from cell counts (>120/genotype) in randomly chosen optical fields of six independent experiments. Urea-induced hypo-osmotic shock caused massive keratin collapse in Plec Ogna/+ but not in Plec +/+ keratinocytes. A trend towards increased K5 IF collapse in untreated Plec Ogna/+ keratinocytes, statistically was not significant. *** P<0.001 (two-way ANOVA with Bonferroni post test). (TIF) [file pgen.1002396.s006.tif]

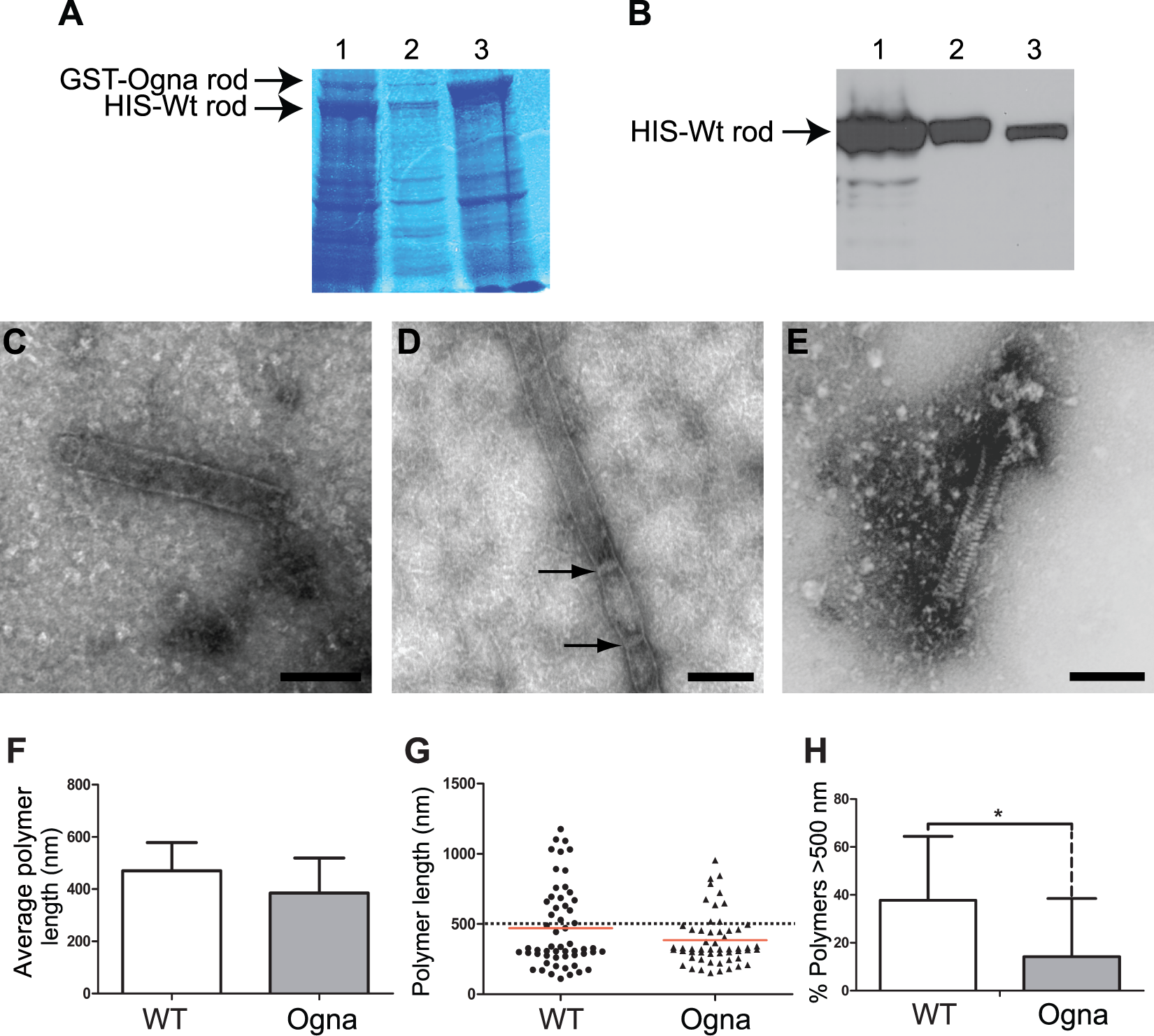

Supplement: Figure S7 — The plectin Ogna rod can hetero-oligomerize with the wild-type rod and can form paracrystalline polymers. (A,B) Protein extracts from Sf9 cells, co-infected with baculoviruses expressing His-tagged wild-type and GST-tagged Ogna-rod versions, were incubated with glutathione Sepharose. The Sepharose-bound GST-Ogna rod and its associated proteins were recovered by centrifugation, washed three times, and eluted with 5x-SDS sample buffer. Eluates were resolved by SDS-8%-PAGE (A) and analyzed by immunoblotting using anti-His-tag antibodies (B). Lanes 1, input; 2, wash fractions; 3, pull-down eluates. Positions of GST- and HIS-tagged rod proteins are indicated. Note, both rods can also form homo-oligomers (Figure 6A) and thus only a fraction of HIS-wild-type rod is recovered in hetero-oligomeric form. (C–E) Electron microscopy of uranyl acetate-stained polymeric (paracrystalline) structures formed from plectin RDs containing the Ogna mutation. pHLH20/Ogna-encoded recombinant versions of plectin's RD were incubated in 50 mM sodium phosphate, pH 7.4, 300 mM NaCl, 170 mM imidazole, and 1 mM PMSF for 1 hour at 37°C before being processed for electron microscopy. Constrictions (D, arrows) of sheet-like structures may represent transition states between flat (collapsed) (C,D) and tube-like (E) structures. Note relative short length of polymers compared to wild-type (Figure 8). Bars, 100 nm. (F–H) Identical concentrations (0.15 mg/ml) of pHLH20/wt- and pHLH20/Ogna-encoded recombinant versions of plectin's RD (Figure 6A), processed in parallel, were adsorbed to 400 mesh grids, and grids subjected to uranyl acetate staining and electron microscopy. Images of 20 RD polymers were generated per protein from randomly selected fields. Three experiments were performed in total. (F) The average polymer length was not significantly different between wild-type (WT) and Ogna RDs, although a trend towards smaller polymer length was noticeable in case of the Ogna RD. Data are shown as mean val [file pgen.1002396.s007.tif]

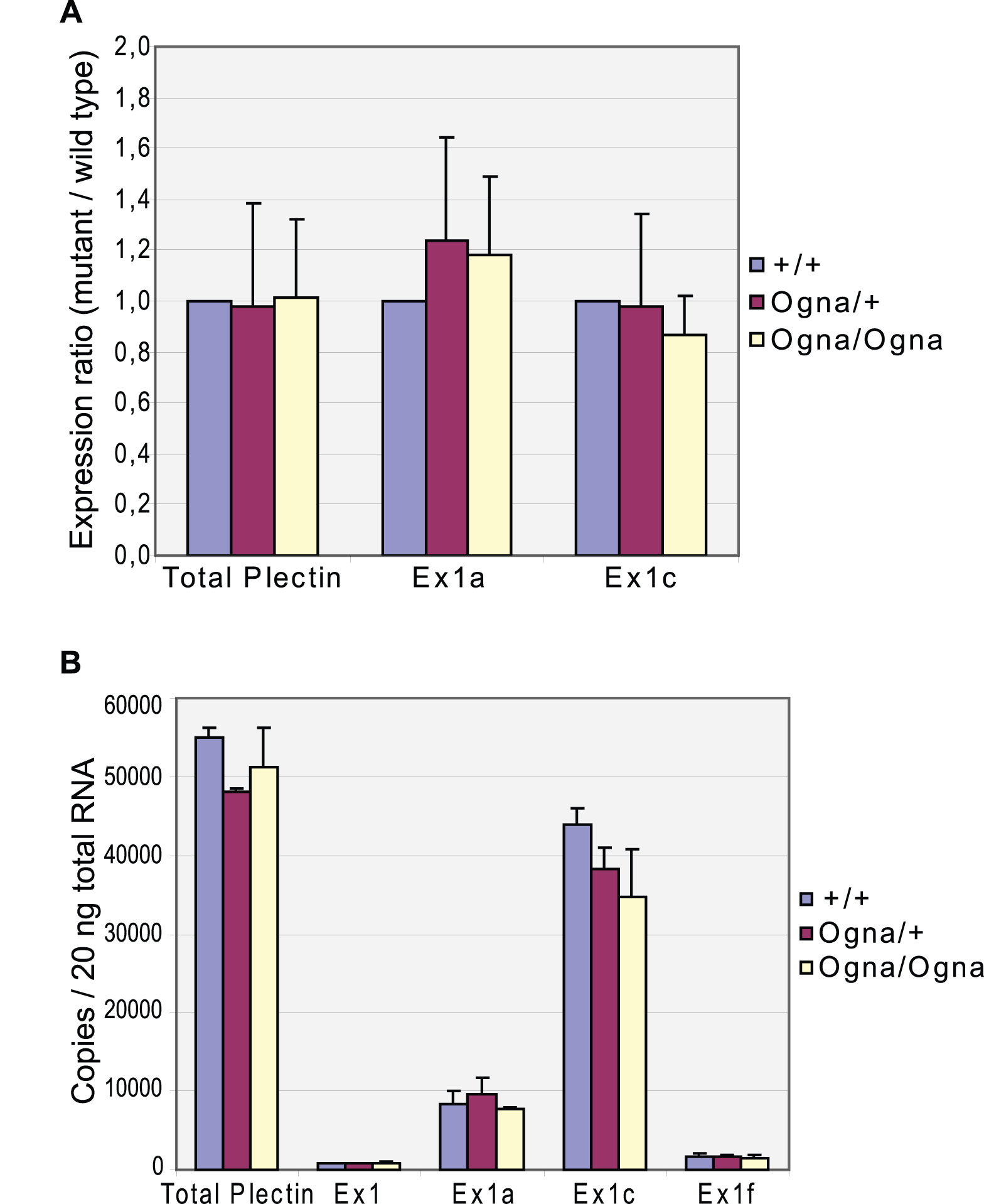

Supplement: Figure S8 — Quantitative analysis of plectin isoform transcripts in epidermal tissues. Total RNA was isolated from epidermis of Plec +/+ and mutant mice using Trizol (Invitrogen). RNA (1 µg) was reverse-transcribed using SuperScript II (Invitrogen). Primer pairs were designed using Primer3 (http://frodo.wi.mit.edu/primer3). The amplicon ranged from 100 to 120 base pairs and spanned an intron. Reactions were performed in 20 µl final volume using SYBR Green I Master Mix (Roche) and processed on a Roche LightCycler 480. Data were analyzed using the software supplied with the instrument. Results originate from three independent experiments with each data point assayed in duplicate. RNA was isolated from two independent samples. (A) Relative quantification. The graph shows the relative expression levels of plectin exons in mutant versus wild-type epidermis. Quantification was done by the method of Pfaffl [78] using the housekeeping gene hypoxanthine guanine phosphoribosyl transferase 1 (HPRT1) for normalization. A ratio of 1 indicates no difference in expression between mutant and wild-type epidermis. (B) Absolute quantification. Results provide insights into the expression of selected plectin isoforms in the epidermis. Transcript copy numbers were calculated using a standard curve based on serial dilutions of the same exons cloned into plasmids. Note that P1c accounts for ∼80% of the total plectin expressed in the epidermis, while P1a represents a lesser fraction. This data are consistent with P1c expression in the basal as well as the suprabasal cell layers while expression of P1a is restricted to basal keratinocytes (see Figure 3, D–I). Columns represent the mean, error bars the SD. (TIF) [file pgen.1002396.s008.tif]

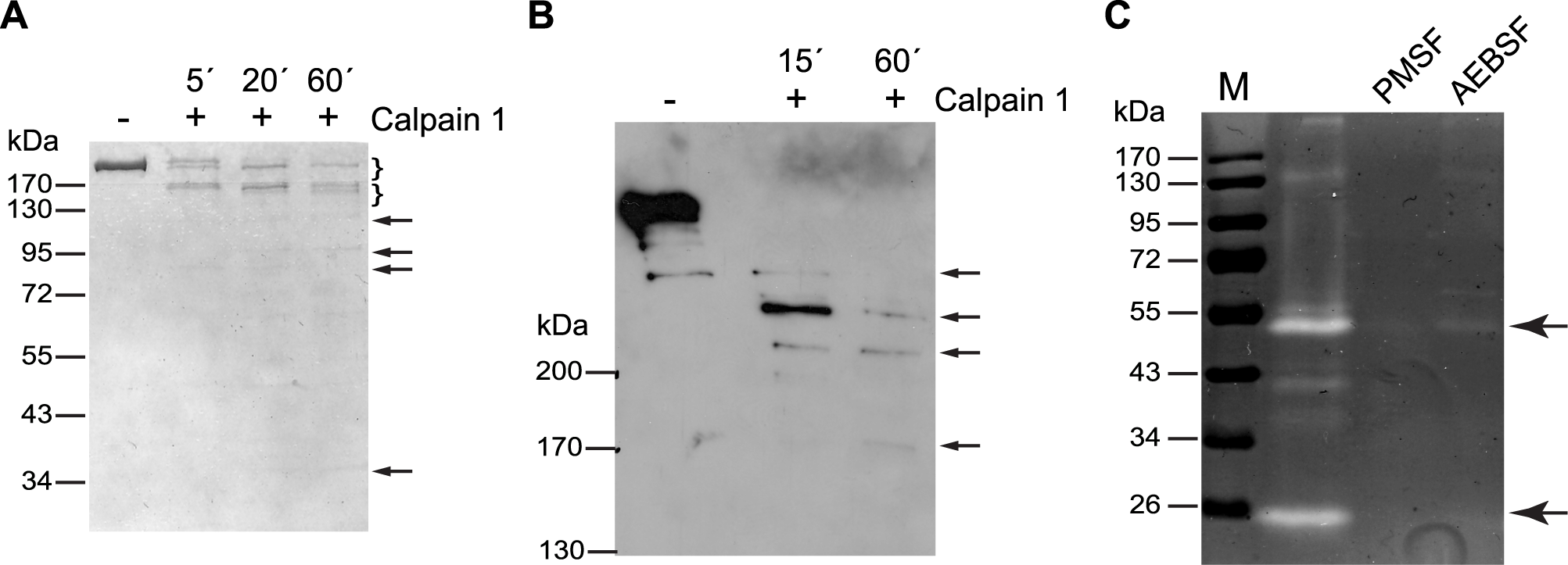

Supplement: Figure S9 — In vitro digestion of plectin by calpain-1 and detection of serine protease activities in epidermal protein extracts. (A) Baculovirus-expressed GST-tagged wild-type rod protein (10 µg) in 30 µl of 20 mM Tris–HCl, pH 7.5, 25 mM NaCl, 1 mM β-mercaptoethanol (solution A) was pre-incubated with 1 mM CaCl2 at 25°C for 5 min before proteolysis was started by adding 10 µl of solution A containing 200 ng of purified human calpain-1 (Sigma-Aldrich), followed by incubation at 25°C for the indicated times. Samples were separated by SDS-10% PAGE, and intact plectin rod protein and cleavage products were detected by Coomassie blue staining. Note the appearance of multiple cleavage products (indicated by arrows and brackets) upon incubation with calpain-1. Similar cleavage patterns were obtained using His-tagged rod protein (data not shown). (B) Native full-length plectin (∼1 µg) purified from glioma C6 cells [79] was subjected to proteolysis as described in (A), except that only 50 ng instead of 200 ng calpain-1 were used. Samples were separated by SDS-5% PAGE, and intact plectin protein and cleavage products (arrows) were detected by immunoblotting using anti-plectin mAb 10F6. (C) Presence of gelatinolytic serine protease activities in epidermal tissue cell extracts analyzed by zymography. Protein extracts were prepared from epidermis as described in Materials and Methods, incubated with either 2 mM PMSF or 5 mM AEBSF (Sigma-Aldrich) to inhibit serine proteases, and then applied to zymography [80] in 12% polyacrylamide gels impregnated with 1 mg/ml gelatin. 15 µg of protein were loaded per lane. Note the presence of strong gelatinolytic activities at ∼52 and ∼24 kDa (arrows), which can be inhibited to a large extent by PMSF and AEBSF, demonstrating that they represent serine proteases. The ∼24 kDa serine protease activity could correspond to trypsin, the expression of which has been demonstrated in basal keratinocytes of skin in situ [81]. (TIF) [file pgen.1002396.s009.tif]

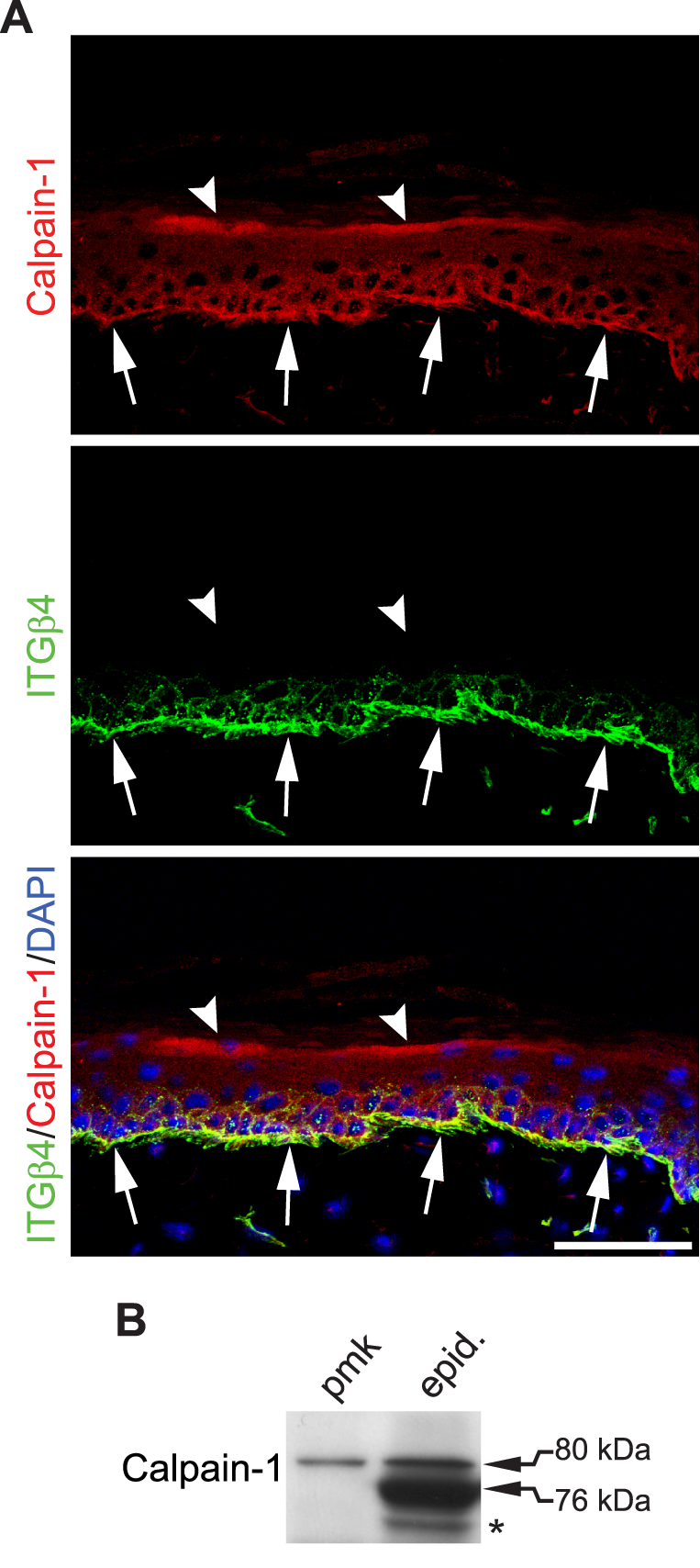

Supplement: Figure S10 — Expression of calpain-1 in mouse skin. (A) Immunolocalization of calpain-1 and ITGβ4 on frozen tail skin sections from adult wild-type mice. Strongest calpain-1 expression is detected in the basal (arrows) and granular (arrowheads) cell layers of the epidermis. Note prominent localization of calpain-1 at the basal cell membrane of basal keratinocytes. Two serial sections of four different wild-type specimens were examined, with no noticeable differences in staining pattern between samples. Bar, 50 µm. (B) Calpain-1 immunoblotting of cell lysates prepared from primary keratinocytes (pmk) (cultured in KGM/0.3) and of tissue extracts prepared from wild-type epidermis (epid.). Note, the 80 kDa (inactive) form of the protease is present in both, keratinocytes and epidermis, whereas the autoproteolytically cleaved (activated) 76 kDa form predominates in the epidermis. Asterisk, protein band of unknown identity. (TIF) [file pgen.1002396.s010.tif]

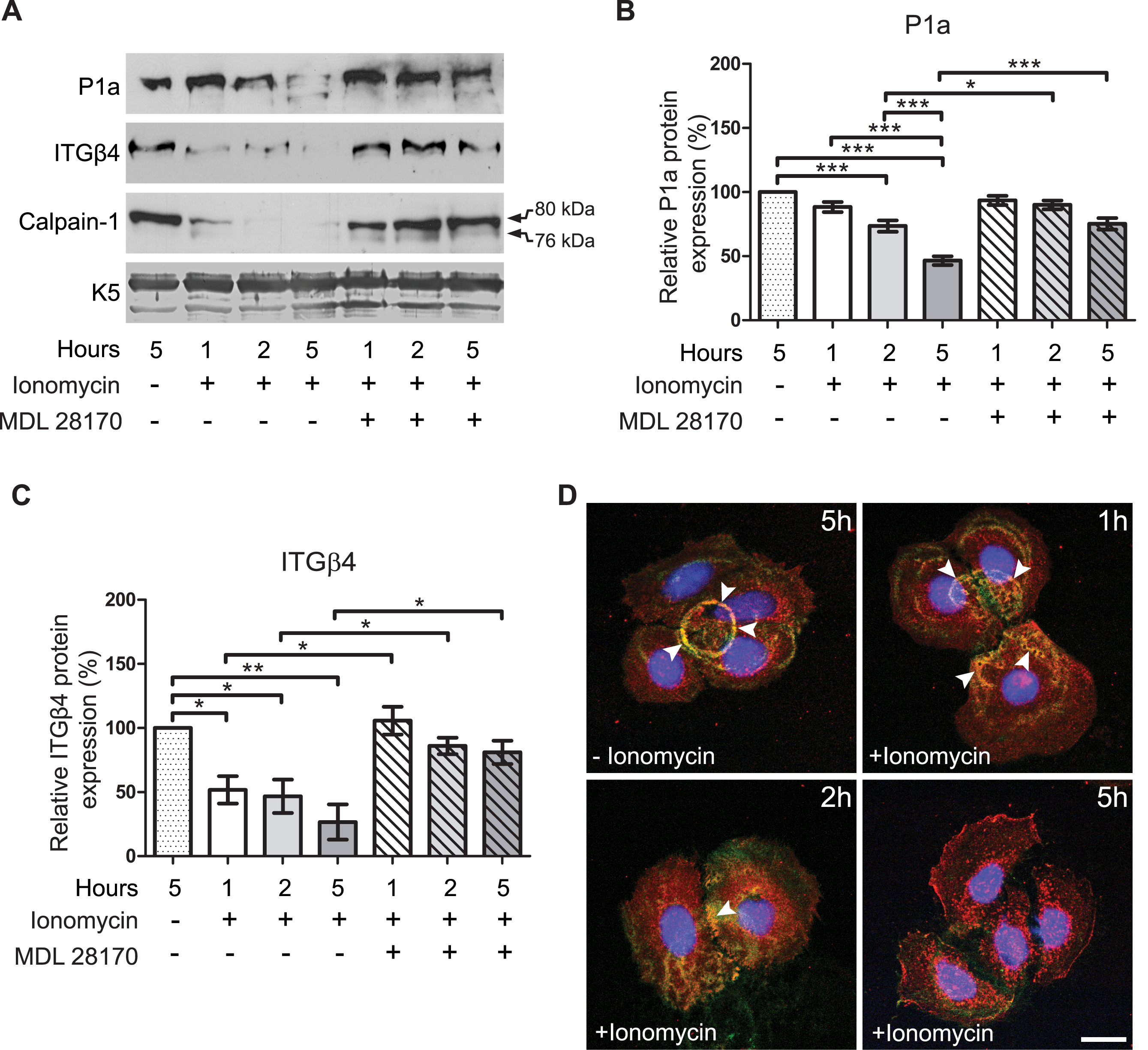

Supplement: Figure S11 — Activation of calpains in cultured keratinocytes leads to degradation of hemidesmosomal proteins. (A–D) Subconfluent cultures of immortalized Plec+/+ keratinocytes were maintained in KGM/0.05, before being switched to KGM supplemented with 1.8 mM CaCl2. Cells were then either left untreated or challenged (for the indicated times) with the Ca2+ ionophore ionomycin (5 µM) with or without the addition of the calpain inhibitor MDL-28170 (50 µM). (A) Immunoblotting of cell lysates using antibodies to proteins indicated. Note strongly decreased levels of calpain-1 due to ionomycin-induced autoproteolytic cleavage (indicative of calpain-1 activation), which was blocked by MDL-28170. Also note gradually decreasing P1a and ITGβ4 levels upon ionomycin challenge, which could be reversed by MDL-28170 treatment. The kinetics of P1a degradation were slower compared to that of ITGβ4, suggesting that P1a was more resistant to calpain-mediated degradation than ITGβ4. The (only) partial inhibition of P1a degradation with MDL-28170 observed after 5 hours of ionomycin treatment likely resulted from the activation of caspases at this timepoint (data not shown). (B,C) Densitometric quantifications of P1a (B) and ITGβ4 (C) protein levels relative to that in control samples (100%) using K5 as loading control. Mean values ±SEM (n = 4) are shown. Statistical significance was demonstrated by one-way ANOVA with Tukey posttest for multiple comparisons (* P<0.05, ** P<0.01, *** P<0.001). (D) Immunolocalization (double labeling) of ITGα6 (green) and plectin (red) in immortalized Plec+/+ keratinocytes with or without ionomycin treatment for the times indicated. Nuclei were stained with DAPI (blue). Composite images were generated from confocal stacks by maximum intensity projections of the three optical sections closest to the substrate level. In untreated keratinocytes, ITGα6 and plectin show codistribution in densely clustered HPCs (arrowheads). After 1 hour of ionomycin challenge, HPCs start to [file pgen.1002396.s011.tif]
