# Supplementary material for: Targeted Proteolysis of Plectin Isoform 1a Accounts for Hemidesmosome Dysfunction in Mice Mimicking the Dominant Skin Blistering Disease EBS-Ogna
Source: PLoS Genet. 2011 Dec 1;7(12):e1002396. doi: 10.1371/journal.pgen.1002396 (PMC3228830; doi:10.1371/journal.pgen.1002396)
Supplement: Table S1 — Primary antibodies used for immunofluorescence microscopy. (DOC) [file pgen.1002396.s012.doc]

**Table S1.** Primary antibodies used for immunofluorescence microscopy.

| **Antigen** | **Antibody** | **Vendor/Catalog#**  **Reference/Name or Clone#** | **Dilution (tissue/cells)** |
| --- | --- | --- | --- |
| Plectin (purified plectin from rat glioma C6 cells) | mouse mAb | Foisner et. al., 1991 /  clone 10F6 | 1:2 (t) |
| Plectin (purified plectin from rat glioma C6 cells) | rabbit antiserum | Wiche and Baker, 1982 /  serum #46 | 1:400 (c) |
| Plectin 1a (N-terminal domain of plectin isoform 1a, protein fragment encoded by exon 1a) | rabbit antiserum,  purified | Rezniczek et al., 1998; Andrä et al., 2003 | 1:100 (t) |
| Plectin 1c (N-terminal domain of plectin isoform 1c, protein fragment encoded by exon 1c) | rabbit antiserum,  purified | Andrä et al., 2003; Fuchs et al., 2009 | 1:200 (t) |
| BPAG1 | mouse mAb | Sugi et al., 1989 /  clone Mab-5E | 1:100 (t) |
| Integrin β4 (C-terminal domain of human ITGb4) | rat mAb | Kennel et al., 1989 /  clone 346-11A | 1:100 (t) |
| Integrin α6 | rat mAb | BD Biosciences /  clone GoH3 | 1:100 (c) |
| Keratin 5 (C-terminal end of mouse keratin 5) | rabbit antiserum,  purified | Covance, Princeton, NJ, USA / PR-B160-P | 1:800 (t)  1:1500 (c) |
| Keratins 5,6,18 (human stratum corneum keratin preparation) | mouse mAb | Dako-Cytomation, Glostrup, Denmark / clone LP34 | 1:100 (c) |
| Desmin | mouse mAb | Dako-Cytomation, Glostrup, Denmark / clone D33 | 1:100 (t) |
| α-Actinin (rabbit skeletal α-actinin) | mouse mAb | Sigma-Aldrich /  Clone EA-53 | 1:800 (t) |
| GFP (purified *a.victoria* GFP) | rabbit antiserum,  purified | Molecular Probes, Eugene, OR, USA | 1:700 (c) |
| Desmoplakin (bovine desmoplakin I + II) | mouse mAbs | Progen, Heidelberg, Germany / clones DP-2.15, DP-2.17, DP-2.20 | undiluted (t) |
| Calpain-1 Large Subunit (synthetic peptide corresponding to human sequence of calpain-1) | rabbit antiserum,  purified | Cell Signaling Technology /  #2556 | 1:50 (t) |
