# Supplementary material for: Targeted Proteolysis of Plectin Isoform 1a Accounts for Hemidesmosome Dysfunction in Mice Mimicking the Dominant Skin Blistering Disease EBS-Ogna
Source: PLoS Genet. 2011 Dec 1;7(12):e1002396. doi: 10.1371/journal.pgen.1002396 (PMC3228830; doi:10.1371/journal.pgen.1002396)
Supplement: Table S2 — Primary antibodies used for immunoblotting. (DOC) [file pgen.1002396.s013.doc]

**Table S2.** Primary antibodies used for immunoblotting.

| **Antigen/Epitope** | **Antibody** | **Vendor/Catalog#**  **Reference/Name or Clone#** | **Dilution** |
| --- | --- | --- | --- |
| Plectin (N-terminal domain of rat plectin, protein fragment encoded by exons 9-12) | rabbit antiserum | Andrä et al., 2003 /  antiserum #9 | 1:3000 |
| Plectin (purified plectin from rat glioma C6 cells) | mouse mAb | Foisner et. al., 19941 /  clone 10F6 | 1:2 |
| Plectin 1a (N-terminal domain of plectin isoform 1a, protein fragment encoded by exon 1a) | rabbit antiserum,  purified | Rezniczek et al., 1998; Andrä et al., 2003 | 1:400 |
| Plectin 1c (N-terminal domain of plectin isoform 1c, protein fragment encoded by exon 1c) | rabbit antiserum,  purified | Andrä et al., 2003; Fuchs et al., 2009 | 1:800 |
| Integrin β4 (N-terminal domain of human ITGb4) | rabbit antiserum,  purified | Santa Cruz Biotechnology, Santa Cruz, CA /  H-101 | 1:200 |
| Keratin 5 (C-terminal end of mouse keratin 5) | rabbit antiserum,  purified | Covance, Princeton, NJ /  PR-B160-P | 1:1000 |
| GAPDH (recombinant fragment of mouse GAPDH) | rabbit antiserum,  purified | Sigma-Aldrich | 1:5000 |
| E-Cadherin (C-terminal end of human E-Cadherin) | mouse mAb | BD Transduction Laboratories, Lexington, KY /  clone LP 36 | 1:2000 |
| GST (purified recombinant GST) | mouse mAb | Sigma-Aldrich /  clone GST-2 | 1:1000 |
| Calpain-1 Large Subunit (synthetic peptide corresponding to human sequence of calpain-1) | rabbit antiserum,  purified | Cell Signaling Technology / #2556 | 1:800 |
